# Investigating the effects of *IDO1*, *PTGS2,* and *TGF-β1* overexpression on immunomodulatory properties of hTERT-MSCs and their extracellular vesicles

**DOI:** 10.1038/s41598-021-87153-7

**Published:** 2021-04-09

**Authors:** Azadeh Haghighitalab, Maryam M. Matin, Ahmad Amin, Shima Minaee, Hamid Reza Bidkhori, Thorsten R. Doeppner, Ahmad Reza Bahrami

**Affiliations:** 1 Department of Biology, Faculty of Science, Ferdowsi University of Mashhad, Mashhad, Khorasan Razavi Iran; 2 Novel Diagnostics and Therapeutics Research Group, Institute of Biotechnology, Ferdowsi University of Mashhad, Mashhad, Iran; 3 Rajaie Cardiovascular, Medical and Research Center, Iran University of Medical Sciences, Tehran, Iran; 4 Department of Cardiovascular Diseases, Razavi Hospital, Mashhad, Iran; 5 Stem Cells and Regenerative Medicine Research Group, Academic Center for Education, Culture and Research (ACECR)-Khorasan Razavi, Mashhad, Iran; 6 Translational Stroke Research Group, Department of Neurology, University Medical School of Göttingen, Göttingen, Germany; 7 Industrial Biotechnology Research Group, Institute of Biotechnology, Ferdowsi University of Mashhad, Mashhad, Iran

**Keywords:** Biotechnology, Cell biology, Immunology, Molecular biology, Stem cells

## Abstract

The therapeutic potential of mesenchymal stem cells (MSCs) is out of the question. Yet, recent drawbacks have resulted in a strategic shift towards the application of MSC-derived cell-free products such as extracellular vesicles (EVs). Recent reports revealed that functional properties of MSCs, including EV secretion patterns, correlate with microenvironmental cues. These findings highlight the urgent need for defining the optimal circumstances for EV preparation. Considering the limitations of primary cells, we employed immortalized cells as an alternative source to prepare therapeutically sufficient EV numbers. Herein, the effects of different conditional environments are explored on human TERT-immortalized MSCs (hTERT-MSCs). The latter were transduced to overexpress *IDO1*, *PTGS2,* and *TGF-β1* transgenes either alone or in combination, and their immunomodulatory properties were analyzed thereafter. Likewise, EVs derived from these various MSCs were extensively characterized. hTERT-MSCs-IDO1 exerted superior inhibitory effects on lymphocytes, significantly more than hTERT-MSCs-IFN-γ. As such, *IDO1* overexpression promoted the immunomodulatory properties of such enriched EVs. Considering the limitations of cell therapy like tumor formation and possible immune responses in the host, the results presented herein might be considered as a feasible model for the induction of immunomodulation in off-the-shelf and cell-free therapeutics, especially for autoimmune diseases.

## Introduction

Mesenchymal stem cells (MSCs) have been widely applied in cell-based regenerative medicine^1,2^. Besides their conventional attribution to regeneration, it is now also evident that MSCs are naturally involved in maintaining immune homeostasis^3^. Immunosuppressive and immune-privileged properties have been reported for these cells^4^, which made them a promising target for exploring their possible therapeutic applications in T-cell-mediated diseases^5,6^. Despite profound outcomes^7^, there are still some controversies in their applications. While MSCs are assessed as safe and efficient based on a vast number of previous pre-clinical and clinical studies, their clinical application is yet limited by the risk of genomic and chromosomal instability acquired during culture, as well as in vivo tumorigenesis and altered functional properties. Moreover, heterogeneity is considered as one of the most important disadvantages of these cells^8,9^.

Immunosuppressive features of MSCs are mainly attributed to their secretion products^10,11^. Among these, indoleamine 2,3-dioxygenase-1 (IDO-1), prostaglandin-E2 (PGE2), nitric oxide (NO), transforming growth factor (TGF)-β1, hepatocyte growth factor (HGF), and interleukin (IL)- 10 have been top listed as the most efficient ones^12–20^. IDO (IDO1) is the rate-limiting enzyme in the tryptophan catabolic pathway^21^ and is regarded as one of the key modulators of acquired immune tolerance^22^. Its immunomodulatory role has been reported in several different studies, particularly during pregnancy^23^, chronic infection^24^, autoimmune diseases^25^, drug resistance in cancer^26,27^, and following transplantation^23^. In addition to other experiments, these observations made a meaningful shift from cell-based therapies to strategies like managing an immunological microenvironment^28–30^. This could be obtained by applying active components of the stem cells’ supernatant, mainly their extracellular vesicles (EVs)^29,31–33^. The EVs have recently been realized as important mediators of intercellular communication processes^34–37^. Hence, EVs are now proposed as exciting means in cell-free therapeutics due to their remarkable properties, including enhanced distribution patterns, higher stability, and lower side-effects^38^. Furthermore, it is also advisable to manipulate parental cells of EVs to provide a tool for cargo transport of enriched desirable agents^39,40^. It seems that EVs are proper candidates to overcome the concerns regarding the therapeutic application of engineered cells. EVs from regular or engineered MSCs are recognized as practical delivery tools in therapeutic programs^41^, even though the consequences of these modifications are still under investigation^42^.

Nevertheless, enhancing the therapeutic efficiency of MSC-derived EVs is currently under investigation worldwide^43^. The utilization of pre-conditioning, genetic engineering, and culture condition modifications could be considered as effective approaches during in vitro expansion of the cells^44,45^. MSCs are prominently in the focus of the exosome-related studies due to their competence for producing remarkable amounts of exosomes. This capacity is not limited to bone marrow MSCs and was also indicated for adipose-derived cells (Ad-MSCs)^46–48^.

In the present study, we first investigated the potential of using immortalized hTERT-MSCs as a proper substitute for primary cells to overcome the problems associated with heterogeneity of the cells, as well as other limitations such as low proliferation capacity and cellular senescence observed during culture of primary mesenchymal stem cells.

Thereafter, we studied secretion patterns of MSCs with regard to EV production. Finally, the cells were further exposed to various treatments or engineered with candidate transgenes to investigate optimal conditions for obtaining a qualified subtype of immunoregulatory MSCs and their corresponding EVs. Thus, the notion here would be empowering the immunomodulatory properties of hTERT-MSCs and their EV counterparts to be introduced as a platform for the management of acute inflammatory status. Induction of allograft tolerance following organ transplantation, reducing the application of immunosuppressive drugs to prevent unfavorable side effects, and solving the endurable and long-lasting problems of patients with autoimmune diseases could be considered as an important perspective of such biological stem cell products.

## Results

### Flow cytometric analysis confirmed desirable immunophenotype of different cell types

**Figure 1:**
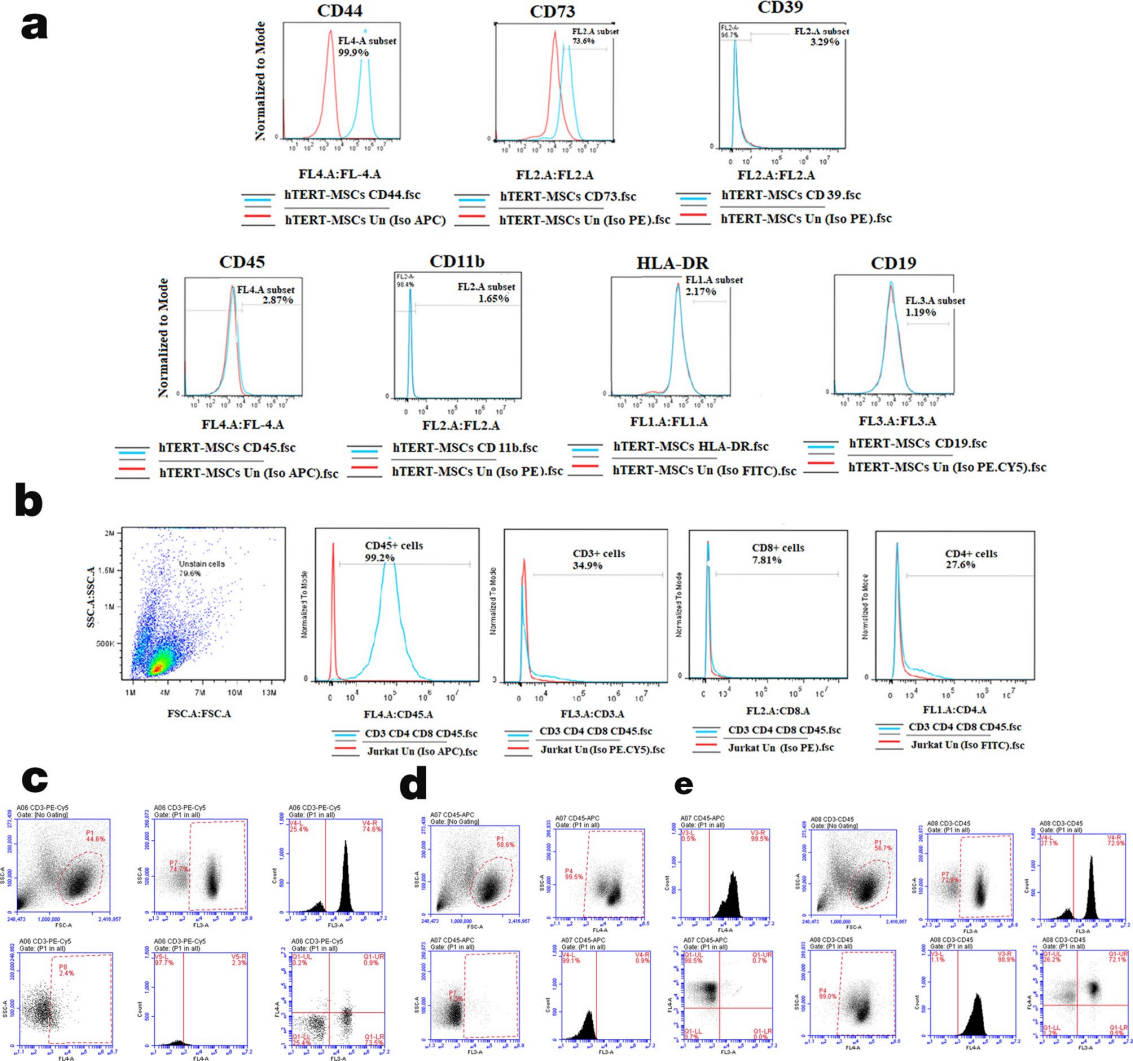
Flow cytometric characterization of different cells. (**a**) hTERT-MSCs: hTERT-MSCs were positive for CD44 and CD73, but they were negative for CD45, CD11b, HLA-DR and CD19 markers. (**b**) Jurkat cells: Most (99.2%) of the Jurkat cells express the hematopoietic cell marker CD45, and 34.9% of the total cell population are CD3^+^. About 90% of these CD3^+^ cells are also positive for CD4 expression. (**c**–**e**) Human PBMCs: About 9% of the cells were positive for CD45 and about 73% of the cells were CD45^+^CD3^+^. Monocytes and platelets were mostly removed by centrifugation steps before analysis. PI staining was applied to remove dead cells from analysis. Data were obtained by BD Accuri C6 and analyzed using FlowJo Software (version 7.6, Becton, Dickinson and Company; 2019).

hTERT-MSCs were characterized for the expression of different molecular markers. As demonstrated in Fig. 1a, these cells follow the same standard expression pattern which is typical of MSCs. As indicated by flow cytometry analysis, around 35% of CD45^+^ Jurkat cells were also positive for the CD3 marker. About 90% of these CD3^+^ cells were concurrently positive for CD4 expression (Fig. 1b). Human PBMCs were mostly composed of CD45^+^/CD3^+^ (72.9%) lymphocytes (Fig. 1c–e).

### The activated anti-inflammatory state of hTERT-MSCs was induced through conditioning or genetic modification strategies

#### IFN-γ induces IDO1 expression in hTERT-MSCs

The chemical treatment with IFN-γ had no adverse effects on the viability of hTERT-MSCs at both 250 U/ml and 500 U/ml concentrations after 72 h, as demonstrated by the MTT assay. We treated the cells with 20 µg/ml poly(I:C), resulting in a decrease in cell viability during the first 24 h, followed by regaining their normal growth rate (Supplementary Fig. S1A). Immunocytochemistry experiments revealed that IFN-γ remarkably induced IDO1 and PTGS2 expressions in the hTERT-MSCs at 72 h post-treatment at the protein level. However, modification in the expression level of the TGF-β1 protein was not considerable in comparison to the untreated cells (Supplementary Fig. S1B). In the case of cells treated with poly(I:C) (20 µg/ml) for 72 h, minor IDO1 expression was detected by fluorescence microscopy at protein level (data not shown). As demonstrated in Supplementary Fig. S1C, Western blot analysis revealed that the main target proteins, IDO1, PTGS2, and TGF-β1 are expressed differentially following treatment with IFN-γ and poly(I:C), in comparison to the untreated cells. Of note, a remarkable increase in protein levels of IDO1 and PTGS2, was evident for the IFN-γ treated cells.

#### hTERT-MSCs express target transgenes

**Figure 2:**
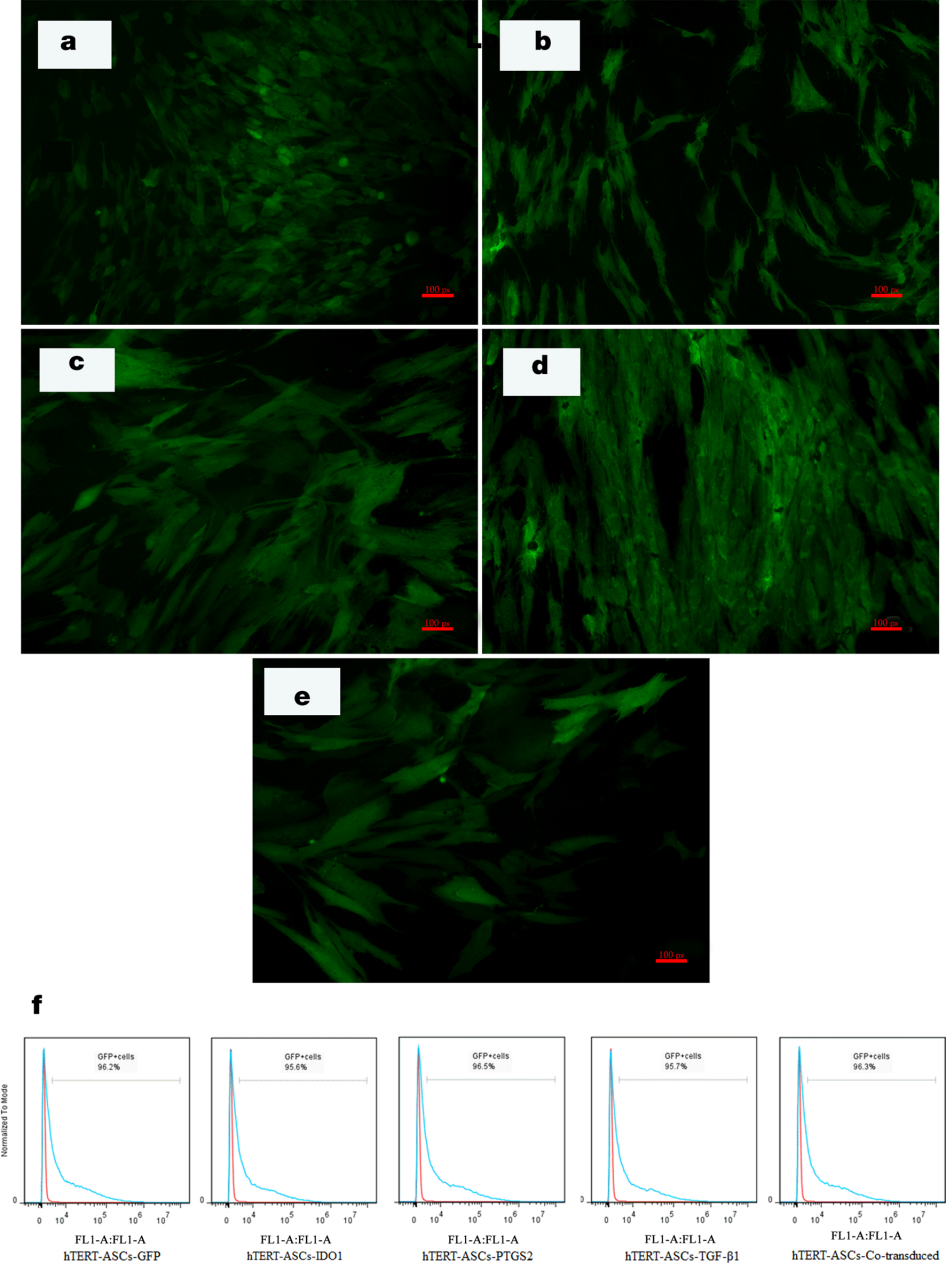
Transduction of hTERT-MSCs with target genes. Fluorescence microscopy analysis of modified hTERT-MSCs (**a**–**e**) represents transduction with *GFP*, *IDO1*, *PTGS2*, and *TGF*-*β1* target genes in addition to transduction with combinatorial viral particles (co-transduced group), respectively (Nikon, Japan, scale bars represent 100 pixel). (**f**) Efficiency assessment of gene transfer into hTERT-MSCs for *GFP*, *IDO1*, *PTGS2*, *TGF*-*β1* or co-transduced cells as evident by flow cytometric detection of green fluorescent protein (Data were obtained by BD Accuri C6 and analyzed using FlowJo Software version 7.6).

HEK293T cells were transfected with the candidate plasmids to overexpress our target genes, including *IDO1*, *PTGS2*, and *TGF-β1* (Supplementary Fig. S2). These genes were short listed based on the results from conditioning experiments and extensive literature review, confirming their functional integrity with immunomodulatory properties of MSCs. Concentrated viral suspensions were applied for infecting the hTERT-MSCs (Fig. 2a–e). As demonstrated (Fig. 2f), in all cases, more than 95 percent of the genetically manipulated cells were positive for GFP expression following 2 days of puromycin selection, confirming that the cells received desirable lentiviral particles in all cases. To confirm the overexpression of target genes following viral transduction, qRT-PCR experiments were performed.

**Figure 3:**
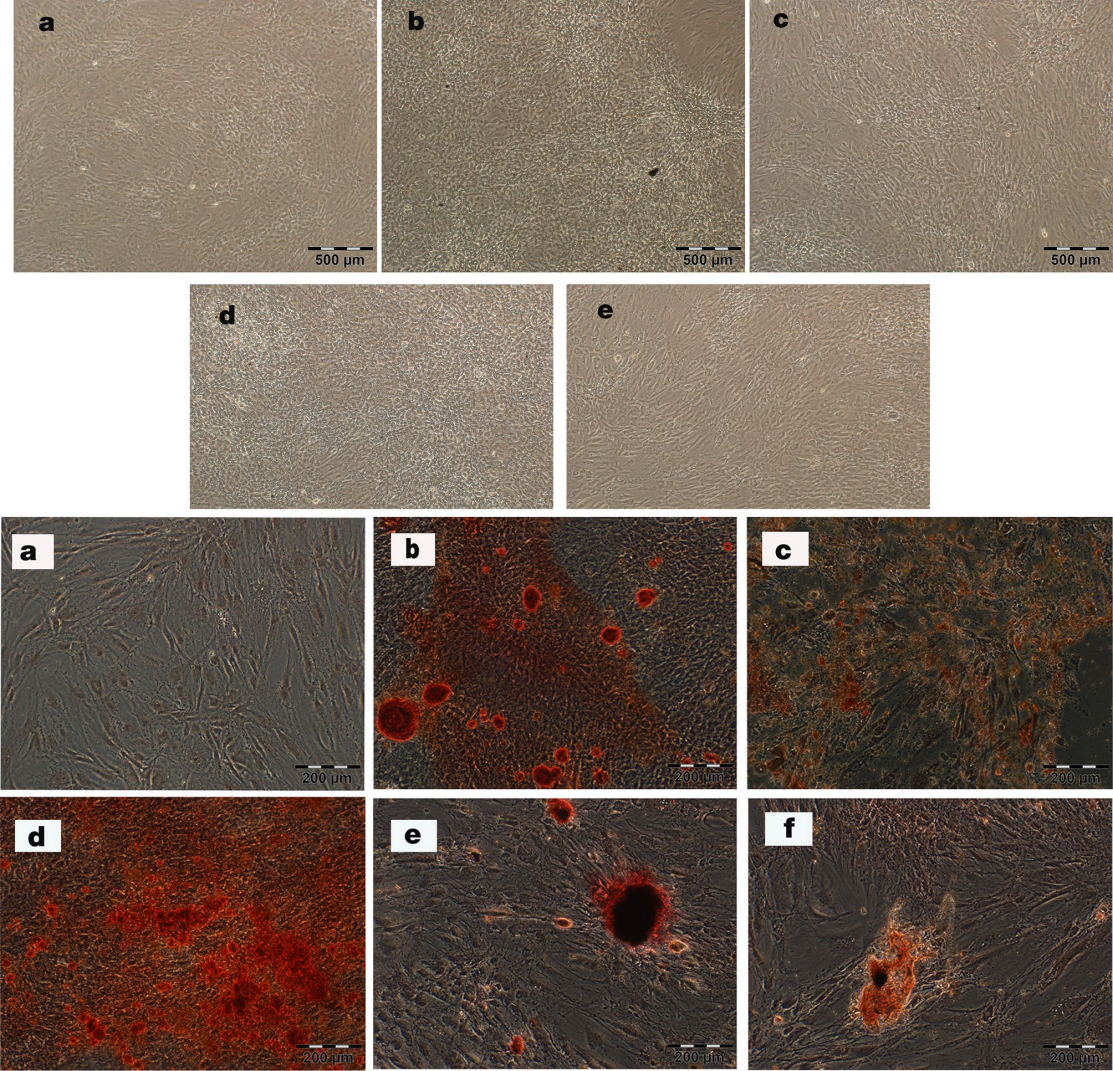
Top; Morphological analysis of hTERT-MSCs. Untreated cells (**a**), hTERT-MSCs-IFN-γ (250 U/ml) (**b**), hTERT-MSCs-poly(I:C) (20 µg/ml) (**c**), hTERT-MSCs-GFP (**d**), and hTERT-MSCs-co-transduced with *IDO1*, *PTGS2* and *TGF-β1* viral particles (**e**) upon reaching confluency (light microscope, Olympus, Japan, scale bars represent 500 µm). Bottom; Osteogenic differentiation of hTERT-MSCs. Untreated/unprimed hTERT-MSCs (**a**) or primed with differentiation inductive medium (**b**) were considered as controls. Cells primed in the permanent presence of IFN-γ (250 U/ml) (**c**), or poly(I:C) (20 µg/ml) (**d**), and following transduction with control GFP (**e**) or co-transduction with *IDO1*, *PTGS2* and *TGF-β1* viral particles (**f**) are shown. Alizarin red S staining is applied for detection of calcification after 21 days of induction (fluorescent microscope, Olympus, Japan, scale bars represent 200 µm).

Microscopic observations indicated that treatment of the cells with IFN-γ (250 U/ml) and poly(I:C) (20 µg/ml) and even their manipulation with the *IDO1*, *PTGS2,* and *TGF-β1* genes did not change the normal morphology of the cells (Fig. 3a–e, *top panel*). To investigate the effects of various modalities on the differentiation potential of hTERT-MSCs towards mesodermal lineages, we cultured these cells in osteogenic induction medium. As shown in Fig. 3 (a–f, *bottom panel*), treatment of the hTERT-MSCs with 20 µg/ml poly(I:C), but not IFN-γ, led to a considerable increase in osteogenic differentiation. The osteogenic differentiation capacity of the cells was not negatively affected by the transgenes in comparison to the cells transduced with the normal GFP expressing vector. As revealed by our flow cytometric analysis, hTERT-MSCs bearing lentiviral constructs retain their mesenchymal origin, as was confirmed by the expression of CD44 and CD73 by more than 95% and 94% of the cells, respectively (Supplementary Fig. S3).

### hTERT-MSCs release desirable amounts of EVs into their conditioned media

The capacity of the hTERT-MSCs for efficient production of EVs was investigated via nanoparticle tracking analysis in addition to the BCA assay. As demonstrated in Supplementary Fig. S4, a remarkable number of EVs with proper size (40–150 nm) and protein concentration are produced by these cells. These EVs were positive for expressing common exosomal markers, including CD63 and TSG101, as approved by Western blot experiments (Supplementary Fig. S4C).

**Figure 4:**
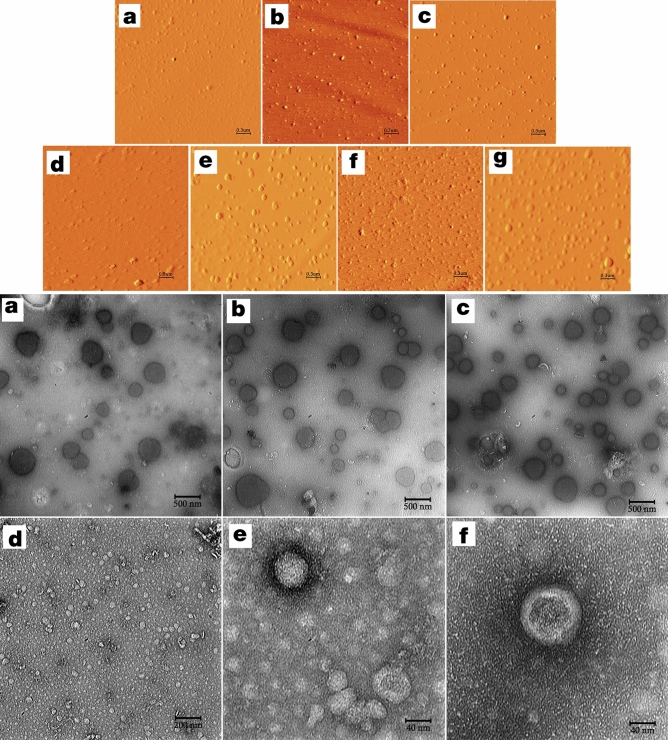
Top; Atomic force microscopy. (**a–c**) represent amplitude images obtained for untreated cells, hTERT-MSCs-IFN-γ (250 U/ml), and hTERT-MSCs-poly(I:C) (20 µg/ml) in scan size range of 3 µm. (**d**–**g**) represent amplitude images obtained for (hTERT-MSCs)-GFP, -IDO1, -PTGS2 and -TGF-β1, respectively. Three different scan size ranges including 3, 5 and 10 µm were applied for the AFM studies. Image processing and analysis were performed by Imager version 1.00 AraResearch Co. Bottom; Electron micrographs from three hTERT-MSCs-GFP derived EV samples (**a–c**) using transmission electron microscopy (Leo 912ab, Zeiss, Germany, 120 kV, Tungsten filament, holy carbon film grid). As demonstrated the vesicle-like particles of different sizes (50–200 nm) as well as lipid bilayer membranes of intact spherical EVs are prominent. (**d–f**) Transmission electron micrographs obtained from PEG-concentrated ultracentrifuge supernatants (20-fold) of the untreated hTERT-MSCs.

Based on characterization data, all EV preparations indicated proper integrity (Fig. 4*top and bottom panels*), size distribution (Supplementary Fig. S4E, F), and polydispersity index (mean PDI 0.2–0.3) in addition to acceptable levels of Zeta potential with proper mobilities (Supplementary Table S1). A heterogeneous surface, obviously detectable for most EV populations in atomic force microscopic images, was an indicator for the presence of proteins in the lipid membrane of the vesicles (Fig. 4*top panel*). The mean EV diameter as measured for 10 particles in each case was equal to 249.81 ± 29.98 (hTERT-MSCs-GFP), 330.72 ± 19.24 (-IDO1), 229 ± 14.58 (-PTGS2), 302.72 ± 19.13 (TGF-β1), and 353.27 ± 11.41 (co-transduced cells) for investigated groups. These particle sizes were in accordance with the ones obtained based on Z-average in particle size analytical experiments. Results of non-contact mode force spectroscopy demonstrated that hTERT-MSCs-IDO1-EVs have the highest level of adhesion/rupture forces (Supplementary Fig. S5).

### Gene expression analysis revealed a differential pattern of expression for a set of innate and adaptive immune-related molecular markers

**Figure 5:**
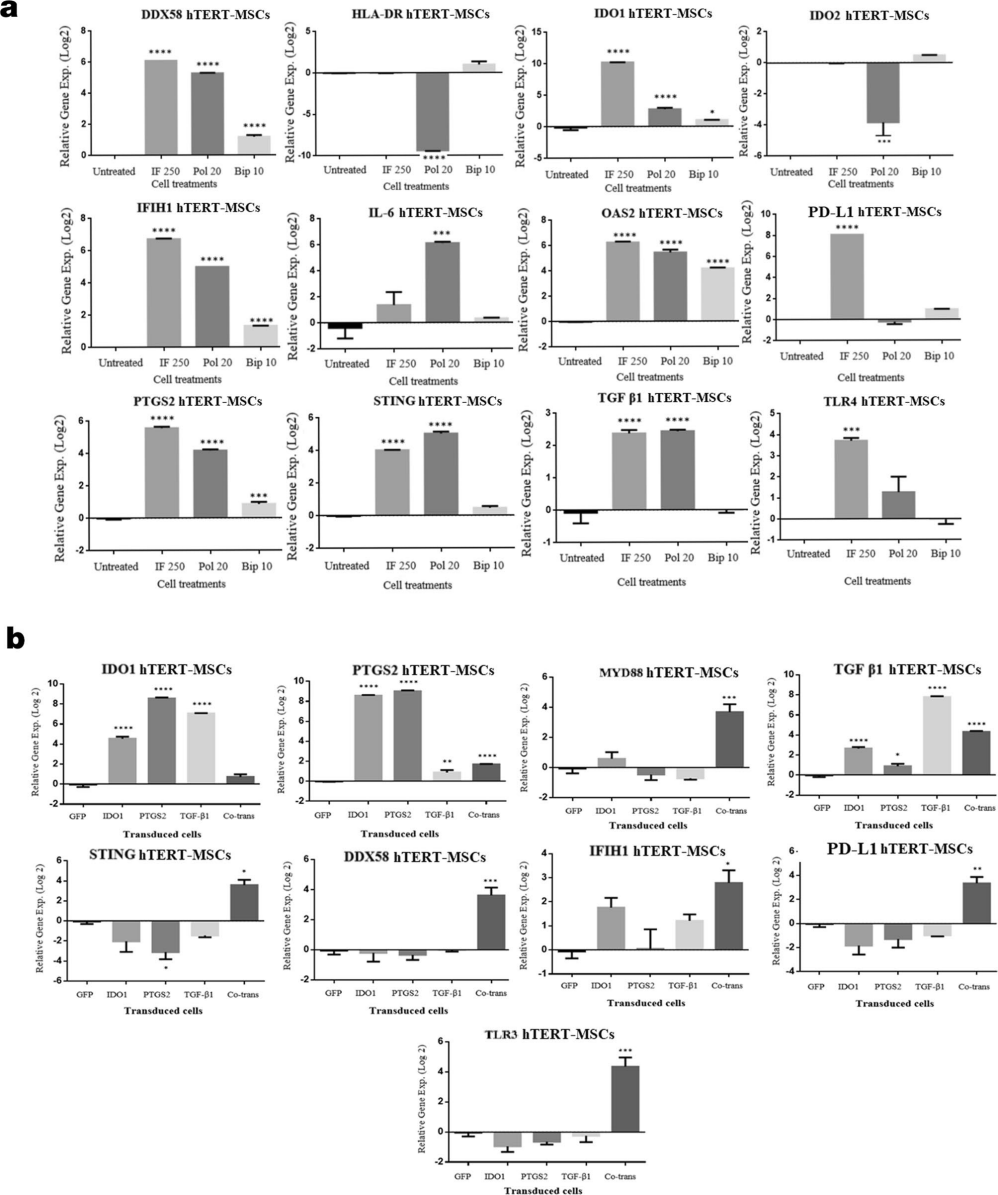
Gene expression analysis. (**a**) Gene expression analysis (mean of independent experiments ± SEM) for hTERT-MSCs following treatment with IFN-γ (250 U/ml, 48 h), poly(I:C) (20 µg/ml, 48 h) and BiP (10 µg/ml, 72 h) in comparison to untreated cells using qRT-PCR analysis. (**b**) Gene expression analysis (mean of independent experiments ± SEM) of the hTERT-MSCs bearing different gene constructs in comparison to GFP transduced cells via qRT-PCR analysis. Among different candidate immune encountered genes, genes depicting statistically significant differences (significance level *p* < 0.05) after modification in comparison to untreated cells/GFP transduced cells are demonstrated. Data analysis was performed by CFX Manager Software (version 1.6) and GraphPad Prism, version 6.00. *p < 0.05, **p < 0.01, ***p < 0.001 and ****p < 0.0001.

Gene expression profiles of hTERT-MSCs were explored following conditioning of the cells with different concentrations of human recombinant IFN-γ and poly(I:C) at various time points (Supplementary Fig. S6A–C). Quantitative RT-PCR experiments revealed that *IDO1* expression is significantly induced with both concentrations of IFN-γ at all the investigated time points, although the highest level of induction was observed for 250 U/ml IFN-γ at 48 h post-treatment. In addition, treating the cells with poly(I:C) (20 µg/ml) led to a minor but significant overexpression of *IDO1* at 48 h (Supplementary Fig. S6B). Likewise, expression of *PTGS2*, *TGF-β1*, and *TDO2* followed the same pattern at this time point. As indicated in Supplementary Fig. S6B*,* a considerable overexpression of the examined genes except for *IDO2* happened 48 h post-treatment. *IDO2*, on the contrary, was downregulated 24 h and 48 h (Supplementary Fig. S6A,B) after treatment but was upregulated in cells treated for 72 h (Supplementary Fig. S6C). Besides, gene expression experiments were performed on the same samples to investigate the expression levels of a set of other genes encountered with immune-related function. As demonstrated in Fig. 5, treating the hTERT-MSCs with both IFN-γ and poly(I:C) resulted in significant upregulation of *DDX-58*, *IFIH1*, *OAS2,* and *STING* genes (Fig. 5a). Moreover, the IFN-γ treatment led to a significant induction in *PD-L1,* and *TLR-4* expression. The transgenic hTERT-MSCs were also applied for gene expression analysis to determine their gene expression profiles (Fig. 5b). These molecular studies revealed that in accordance to the cells treated with IFN-γ or poly(I:C), the expression of *MYD88*, *STING*, *DDX-58*, *IFIH1,* and *PD-L1* was significantly induced in hTERT-MSCs-co-transduced cells. While in hTERT-MSCs transduced with each of the transgenes individually, we did not observe any statistically significant modifications in the expression levels of these genes.

### hTERT-MSCs and their cell-free products are immunosuppressive

#### hTERT-MSCs exert immunosuppressive features under different circumstances

**Figure 6:**
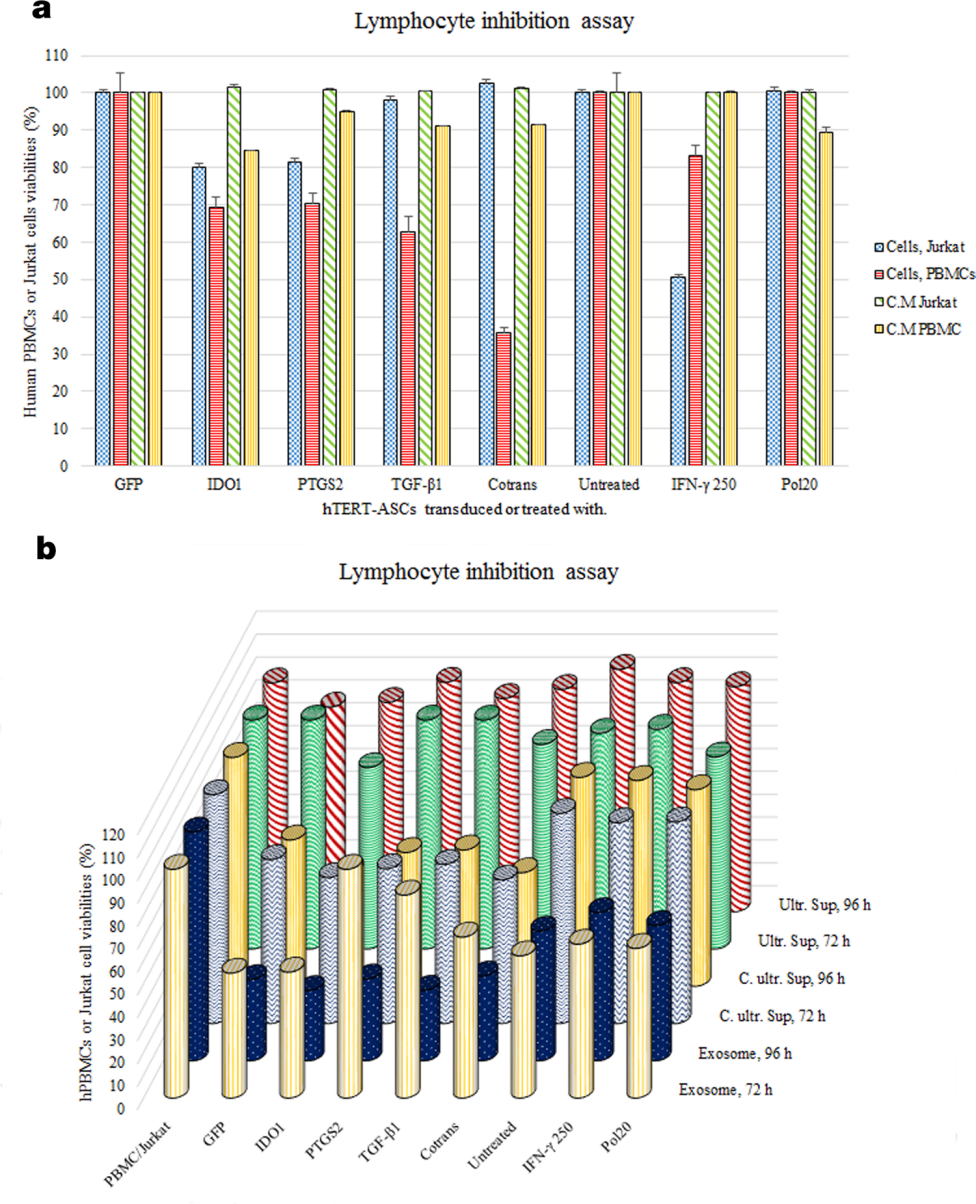
Lymphocyte inhibition assay. (**a**) Lymphocyte inhibition assay was performed following three days of co-culture experiments between PBMCs or Jurkat cells and hTERT-MSCs transduced with different viral particles or treated with interferon gamma (250 U/ml) or poly(I:C) (20 µg/ml). As demonstrated, some experiments were performed for 4-day conditioned media of the cells. Relative ODs (450 nm) in each group were read following 4 h incubation of Orangu dye with hPBMCs by an ELISA reader (Awareness Technology, USA). Data are represented as mean of viabilities ± SEM. (**b**) Functional analysis of cell-free products from the hTERT-MSCs. Lymphocyte inhibition assay experiments were performed for exosomes, PEG-concentrated ultracentrifuge supernatants and ultracentrifuge supernatants without any modification in the permanent presence of 3 µg/ml PHA as proliferation stimulant. 72 h and 96 h co-cultures were applied for functional analysis based on the detection of viable cells via sensitive Orangu dye. All experiments were performed against human PBMCs except for the ultracentrifuge supernatant which was performed against Jurkat cells. During all co-culture experiments we applied the hPBMCs/Jurkat cells to MSCs ratio as 10:1. For cell-free products 100 µg of the products were added to the culture media of hPBMCs/Jurkat cells (10^6^ cells/well). Data represent mean of three independent experiments. Relative ODs (450 nm) in each group were read by an ELISA reader (Awareness Technology, USA) following 4 h incubation.

The functional analysis using lymphocyte inhibition assay (colorimetric assay, Orangu dye) revealed that the hTERT-MSCs inhibited the proliferation of human PBMCs by 17% following treatment with 250 U/ml IFN-γ. This was more efficient following the transduction of the cells with viral particles carrying the gene constructs of *IDO1*, *PTGS2,* or *TGF-β*1 genes, as the mean inhibition rates were scored as 31%, 30%, and 38%, respectively (Supplementary Table S2). However, the hTERT-MSCs co-transduced with all three viral particles showed the highest level of lymphocyte proliferation inhibition (more than 60%) (Fig. 6a*, red columns*). As demonstrated, hTERT-MSCs-IFN-γ also exert significant inhibitory effects against Jurkat cells (50%), while similar effects against Jurkat cells were only observed for *IDO1* and *PTGS2* transduced cells, though to a lesser extent (Fig. 6a*, blue columns*). Regarding the conditioned medium of the cells applied for functional analysis, no inhibitory effects were observed against Jurkat cells (Fig. 6a*, green columns*). However, anti-proliferative effects of virally transduced cells against human PBMCs were evident when compared to GFP transduced cells. These inhibitory effects were more prominent (~ 20%) in the case of *IDO1* transduced cells (Fig. 6a*, yellow columns*).

**Figure 7:**
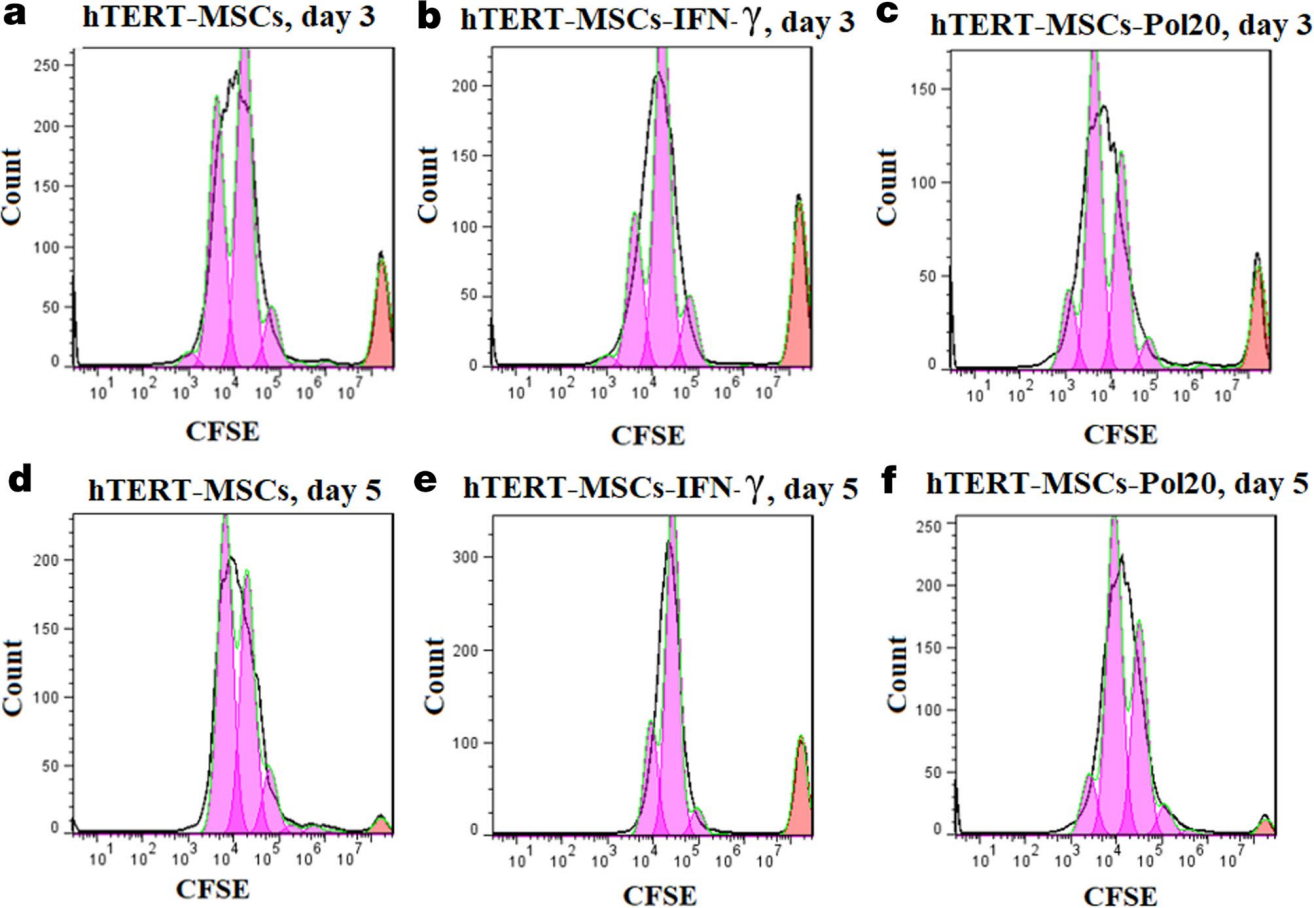
Lymphocyte inhibition assay (CFSE analysis). Results from flow cytometric analysis of the inhibitory effects of hTERT-MSCs against Jurkat cells following 3 to 5 days of co-culture experiments. (**a**,**d**) untreated hTERT-MSCs, (**b**,**e**) IFN-γ (250 U/ml)-primed and (**c**,**f**) poly(I:C) (20 µg/ml)-primed cells after 72 h and 120 h of culture, respectively. 3 µg/ml of PHA was applied as cell proliferation stimulant for Jurkat cells. IFN-γ priming of the cells resulted in lower percentage of divided cells and decreased amounts of division index in both investigated time periods in comparison to untreated cells (Jurkat cells: MSCs ratio 10:1). Data were obtained by BD Accuri C6 and analyzed using FlowJo Software (version 7.6).

**Figure 8:**
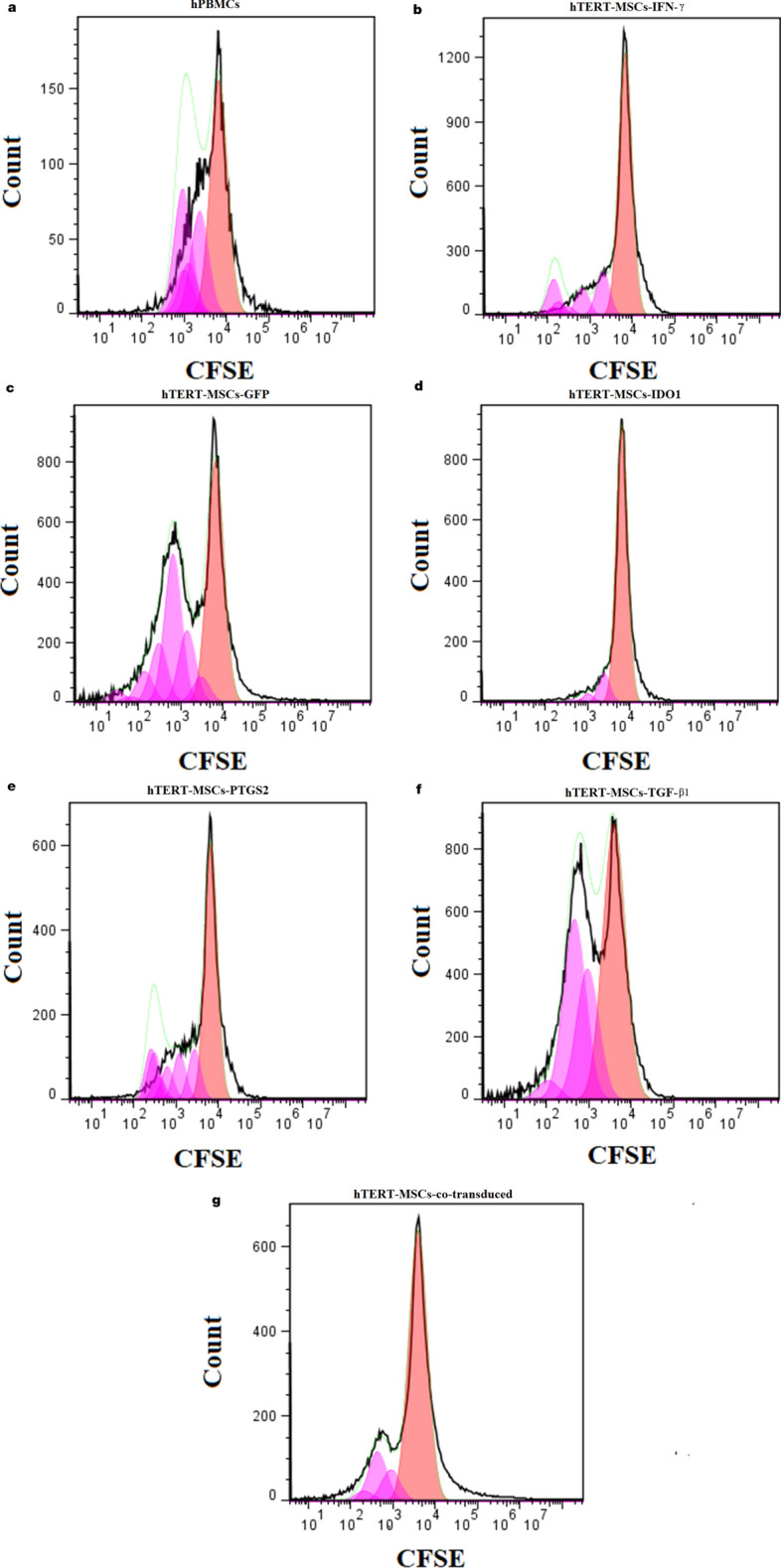
Lymphocyte inhibition assay (CFSE analysis). Results from flow cytometric analysis of the inhibitory effects of hTERT-MSCs against human PBMCs following 2 days of co-culture experiments. CFSE-labelled human PBMCs cultured (**a**) in the absence of any cells or in the presence of (**b**) hTERT-MSCs-IFN-γ (250 U/ml), (**c**) hTERT-MSCs-GFP, (**d**) hTERT-MSCs-IDO1, (**e**) hTERT-MSCs-PTGS2, (**f**) hTERT-MSCs-TGF-β1 and (**g**) hTERT-MSCs-co-transduced. As shown hTERT-MSCs-IDO1 demonstrated the highest level of lymphocyte inhibition followed by hTERT-MSCs-co-transduced (PBMCs: MSCs ratio 10:1). Data were obtained by BD Accuri C6 and analyzed using FlowJo Software (version 7.6).

Inhibitory effects of IFN-γ treated hTERT-MSCs against Jurkat cells were also confirmed during flow cytometric CFSE analysis for PHA stimulated cells (the default number of peaks: 8). Inhibitory effects of IFN-γ treated cells led to a remarkable decrease of divided cells (%), from 17 to 10.6% on day 3 and 41.3 to 3.1% on day 5, for primed cells in comparison to untreated cells, respectively (Fig. 7). Moreover, proliferation and division indices of 4.84 and 0.63 were respectively obtained for PBMCs cultured with IFN-γ-primed cells compared to untreated cells (5.28 and 2.18) on day 5. Similar immunosuppressive effects were also confirmed for IFN-γ treated cells (Precursor frequency or %Divided: 10.9%) in comparison to untreated cells (%Divided: 24.9%) via flow cytometric approach on day 2 of co-culture experiments with hPBMCs (Fig. 8a,b). CFSE labeling also confirmed the improved immunosuppressive features of all virally transduced cells in comparison to GFP transduced cells. Percent divided (%Divided), division index (Div. Index), proliferation index (Prol. Index) and Peak CVs obtained for different treatments following 2 days of co-culture experiments were as follows respectively: hTERT-MSCs-GFP (18.4%, 0.42, 2.31, 8.43), hTERT-MSCs-IDO1 (2.98%, 0.06, 2.23, 5.81), hTERT-MSCs-PTGS2 (14.8%, 0.25, 1.73, 6.35), hTERT-MSCs-TGF-β1 (16.8%, 0.41, 2.44, 12.1) and hTERT-MSCs-co-transduced (5.15%, 0.13, 2.52, 9.99) (Fig. 8c–g). These data from colorimetric and flow cytometric analyses indicated the superiority of *IDO1* transduced cells compared to other virally transduced cells for in vitro induction of lymphocyte inhibition.

#### EVs from primed/genetically modified hTERT-MSCs exert in vitro immunosuppressive properties

As inhibitory effects were revealed for all transduced cells, the possibility of mimicking these effects by their extracellular counterparts, emphasizing on EVs was also explored. Likewise their parent cells, regardless of the modification target, EVs from transduced cells exhibited the most inhibitory effects in comparison to EVs from unconditioned, or IFN-γ- and poly(I:C)-primed hTERT-MSCs (Supplementary Table S3). Analysis of the colorimetric Orangu assay revealed 70% inhibition of the human PBMCs proliferation when exposed with EVs derived from the aforementioned transduced cells (*IDO1*, *PTGS2*, and *TGF-β1*) compared to the PBMCs in the absence of any EVs (Fig. 6b*, Exosomes, 96 h co-culture*). However, this rate difference was just significant in the case of *IDO1* EVs against hTERT-MSCs-GFP EVs after 96 h (*p* < 0/0001). It means that despite the superior immunomodulatory properties of GFP transduced cells in comparison to untreated or primed cells, the expression of *IDO1* in EV producer cells enhanced the immunomodulatory properties of their descendent EVs by 13.54%, which is a considerable amount (Supplementary Fig. S7). This is achieved by considering the mean viability of the PBMCs in the presence of hTERT-MSCs-GFP EVs equal to 100%. Then, the mean inhibitory effects for IDO1 EVs will be equal to 86.49%.

Our data reveals that the second least inhibitory effect of the exosomes on PBMC proliferation belongs to those extracted from the cells which were transduced with GFP constructs. This inhibition is in line with some previous reports which postulated that GFP protein expression can cause T-cell mediated immunogenicity and apoptosis^49^. Meanwhile such base line effect is apparently neutralized in treatments with exosomes of the *PTGS2* and *TGF-β1* constructs rather than *IDO1* for unknown reasons. One could argue that they may load less GFP protein into the exosomes or cover its functional negative effects. This remains as an open question to be investigated with further experiments. This however does not change the fact that IDO1 carrying exosomes exert the highest level of PBMC inhibition.

Inhibitory effects of the ultracentrifuge-supernatants as negative controls were also explored against Jurkat cells during our experiments. Although some significant inhibitory effects were observed for the ultracentrifuge-supernatants obtained from the cells transduced with *IDO1* (21%) and co-transduced (11%) cells after 72 h, such effects were demolished in the medium after 96 h in the presence of PHA (Fig. 6b).

Inhibitory effects of EVs from modified hTERT-MSCs against allogenic human PBMCs were also confirmed via flow cytometric approach. As revealed by the CFSE cell inhibition assay, peak ratios and proliferation indices were decreased for EVs from virally transduced or IFN-γ primed cells in comparison to EVs from hTERT-MSCs-GFP. Proliferation indices for different treatments were recorded as GFP-EVs (2.68), IDO1-EVs (2.31), PTGS2-EVs (1.47), TGF-β1-EVs (2.28), and co-trans-EVs (2.62). It was accompanied by a higher number of the parent cells left undivided in the presence of EVs from *IDO1*, *TGF-β1,* and IFN-γ groups.

## Discussion

Characteristics such as easy accessibility, multi-lineage differentiation capacity, immunoregulatory features, directed tissue homing, and less ethical concerns have led to an extensive application of MSCs in regenerative medicine. Nevertheless, translational results to apply MSCs in clinical settings are still insufficient^50^. The reason of the latter lies in heterogeneous phenotypes of MSCs and high clearance rates of grafted MSCs within injured organs and tissues^51^. Interestingly, the biological functions of MSCs depend on their surrounding microenvironment, yielding defined paracrine effects for which EVs play a significant role^52^. MSCs have a remarkable capacity for transferring their mitochondria and efficient release of exosomes^53–58^. EVs are crucial for intercellular communication processes between immune-competent and non-competent cells^59, 60^, and modifying immune responses within the host organ^52,61–63^. Although the precise molecular mechanisms by which MSC-derived EVs regulate the immune responses are still unknown, evidence suggests that MSCs are able to transfer various cargos such as miRNA and others by EVs.

As the biological properties of MSCs depend on their cellular microenvironment, the characteristics of EVs also depend on their parental cells^64^. Hence, various pre-conditioning techniques or modification of MSC expression profiles have recently been regarded to enrich EVs with desirable features^65–69^. The present study strived first to improve the immunomodulatory capacity of MSCs and their EV derivatives thereafter. As such, the experimental paradigm included priming of hTERT-MSCs with various agents or the ectopic expression of different genes in these cells as described in the materials and methods. EV enrichment was performed from these different host cells. The treated cells or their derivatives from both experiments were subjected to functional analysis to evaluate their immunoregulatory properties.

Herein, we used hTERT-MSCs as a model cell line due to the prominent immunoregulatory properties of adipose-derived MSCs compared to other sources of MSCs^70–74^. Furthermore, these cells have a normal karyotype and differentiation capacity and do not show any tumorigenic effects in vivo^75^. In line with this, hTERT-immortalization was previously proposed as a feasible way of producing cell lines with an amenable ability for producing therapeutic EVs^76–78^.

For the first time, a report by Krampera attributed IFN-γ priming of bone marrow-derived MSCs to the enhancement of immunomodulatory proteins, mainly IDO1^79^. Similar observations were also described for poly(I:C), a well-known analog of dsRNA^80–82^, and BiP/Glucose-Regulated Protein 78 (GRP78)^83–90^, albeit IFN-γ has been investigated most intensively. The ability of type II interferon for inducing the expression of immunoregulatory encountered genes, including *IDO1*, in MSCs from different sources was previously reviewed^91,92^. Our quantitative RT-PCR experiments performed to elucidate possible effects of BiP on the expression levels of some immune-related genes, for the first time, indicated its ability for the induction of several innate and adaptive effector gene expressions in the hTERT-MSCs. A concentration of 10 µg/ml BiP can recapitulate, to some extent, the molecular profile of IFN-γ-treated cells at 72 h.

Based on the analyses performed in the present study, the increased immunomodulatory capacity of hTERT-MSCs was in the same order as primary cultures of MSCs upon treatment with IFN-γ. Furthermore, the Western blot experiments confirmed the existence of the IDO1 protein in EVs from hTERT-MSCs-IFN-γ, but not for the untreated or poly(I:C)-treated cell-derived EVs. This is in line with a recent report that exosomes isolated from IFN-γ primed BM-MSCs were enriched with full-length *IDO1* transcripts. Interestingly, exosomal transfer of the *IDO1* transcripts toward PBMCs resulted in an increased expression of IDO1 in these recipient cells^39^.

The importance of IDO1 among other soluble factors released to the conditioned media of primary cultures of MSCs is already known^93,94^. In line with the interesting results obtained from treatment of hTERT-MSCs with IFN-γ, human *IDO1*, *PTGS2,* and *TGF-β1* genes were shortlisted for ectopic expression in these cells. These were recognized as soluble factors secreted by MSCs and are responsible for some parts of their functional properties^95^.

Molecular analysis performed on the genetically modified hTERT-MSCs in this study revealed remarkable overexpression of the transgenes with downstream functional consequences. Ectopic expression of *IDO1*, *PTGS2,* and *TGF-β1*, individually or in combination, significantly increased the immunomodulatory properties of hTERT-MSCs as demonstrated by the lymphocyte inhibition assay. These antiproliferative effects against human PBMCs were more efficient in comparison to what was obtained by priming the cells with IFN-γ.

Many authors suggest that MSCs possess an intrinsic capacity for inducing innate and adaptive immune system players to create a tolerogenic environment^96–101^. Based on the molecular and functional analyses performed in the present study, this ability is also confirmed for hTERT-MSCs licensed by IFN-γ or genetically engineered cells to concurrently overexpress *IDO1*, *PTGS2,* and *TGF-β1* in the co-transduced cells.

The expression levels of different genes were investigated in the present study to compare the molecular profile of hTERT-MSCs following the application of priming/gene manipulation schedules. IDO1, IDO2, and TDO2 are three enzymes that are encountered in the tryptophan degradation pathway, among which IDO1 is the most studied one due to its unique properties. As *IDO2* and *TDO2* are also involved in regulating immune responses^102,103^, we investigated their expression patterns in primed and/or modified cells to monitor their modifications^104,105^. In the case of the IFN-γ (250 U/ml, 48 h)-primed cells, which were applied as the positive control during our experiments, concurrent overexpression of the main target genes was observed in the absence of *IDO2* or *TDO2* with overlapping functions^106^. Although IDO1 and its homolog IDO2 are encoded by linked genes, their immunoregulatory features seem to be completely different. Whereas IDO1 mediates T-cell responses in the benefit of anti-inflammation, IDO2 mediates B-cell responses towards a pro-inflammatory phenotype^107^.

From the mechanistic point of view, IDO1-mediated immunoregulation is attributed to the establishment of a local tryptophan-deficient microenvironment, initiating a metabolic stress status that activates GCN2 kinase and mTOR pathways. Consequently, these events lead to T cell anergy. IDO1 also mediates the activation of regulatory T cells to create an in vivo immunomodulatory status.

IFITM and OAS families are classified as ISGs, which have prominent roles during the antiviral immune defense. The ISGs themselves are classified into antiviral effectors and regulators of type I IFN signaling^108^. ISG15, 2′–5′-oligoadenylate synthetase (OAS) is one of the most studied ISGs that is a ubiquitin-like protein. It is found in both intracellular and extracellular spaces as a free or covalently protein-conjugated molecule^109^. ISG15 is a critical part of the antiviral defense mechanism in mice^110^, whereas such a role is not confirmed in humans. However, ISG15 plays a role in anti-mycobacterial immunity in humans^111^.

Different pathways are involved in cytokine signaling based on the regulation of the immune system, including interferon-alpha/beta signaling, interferon-gamma signaling, interleukin-1 family signaling, interleukin-10 signaling, and the antiviral mechanism by IFN-stimulated genes (ISG15 antiviral mechanism)^112^. DDX-58, an innate immune receptor, is involved in DDX-58/IFIH1-mediated induction of interferon-alpha/beta and antiviral mechanism triggered by IFN-stimulated genes. IFIH1 (MDA-5) is another innate immune receptor that plays a prominent role in mediating innate immune responses via recognition of RNA metabolites produced by RNase L (www.genecards.org). *IFIH1* expression leads to the activation of interferon cascade, whereas *OAS2* gene expression is induced by both type I and type II interferons^113^. Upregulation of *IDO1*, *IFIH1,* and *OAS2* was reported following infectious diseases^112^. STING is a pattern recognition receptor whose alterations were investigated due to its role in autoimmune diseases and innate immune responses following the detection of cytoplasmic DNA (www.genecards.org). Detailed molecular mechanisms encountered with STING functions during inflammation, infection, and cancer are fully described previously^114^. HLA-DR expression as an MHC class II cell surface receptor was also investigated to compare the immunomodulatory status of the cells. In a study performed by Mao and coworkers, expression levels of *Il-6*, *Il-10*, *Ccl2*, *Ccl5*, *TGF-β*, *Nos-2*, *TSG-6*, *Mpges-2,* and *Cox-2* were investigated as genes with immunoregulatory functions in MSCs following microencapsulation^115^. It was also demonstrated that IDO1, HGF, PD-L1, and IL-6 were induced following the secretion of IFN-γ by activated T-cells. These factors play a prominent role in recapitulating the proper microenvironment for MSCs to express their immunoregulatory functions^93^.

It was also concluded that 104 peptides, including some proteins with anti-inflammatory functions such as Gal-1 and PD-L1^116–121^ were uniquely enriched in EV preparations from MSCs primed with IFN-γ. This study was the first experiment that confirmed the prominent role of IDO1 in mediating EVs' therapeutic efficacy in vivo using a mouse model of experimental autoimmune encephalomyelitis (EAE)^39^.

Significant results were also observed for EVs extracted from the conditioned media of all genetically modified hTERT-MSCs in comparison to EVs from control or even primed cells with IFN-γ or poly(I:C). It is noted that *IDO1* significantly improved the immunosuppressive features of cells, their conditioned media, and EV preparations in comparison to their equivalents from mock-transfected cells. In line with our findings, previous publications have demonstrated the improvement in immunomodulatory properties of different cell types following the ectopic expression of *IDO1*, which is widely accepted as one of the main playing factors in MSC immunomodulation^22,71,122,123^. In the present study, we demonstrated the superior properties of EVs derived from hTERT-MSCs-IDO1 from different aspects to benefit their functional immunosuppressive properties.

This statistically significant difference in the functional properties of IDO1-derived cell-free products, could be attributed to the appropriate size of EVs, their higher stability, and proper biomechanical properties. The remarkable increase of IDO1 in the conditioned media of the hTERT-MSCs-IDO1 and its transfer/uptake by PBMCs could be considered as effective mechanisms facilitating the superior immunoregulatory properties of the cells. As revealed by qRT-PCR experiments the conditioned media of IDO1-transduced cells was enriched (2^28^-fold change) with *IDO1* transcript in comparison to GFP controls. It was previously proposed that IDO1 can define a local immunosuppressed microenvironment via its catalytic activity and fine tuning of active immune cells' balance. This includes mainly cytotoxic and regulatory T cells, B cells, and natural killer cells to benefit an immune-balanced microenvironment^124,125^.

It is now vastly believed that EVs are instrumental in the packaging and delivery of the communicating molecules. Developing protocols for a generation of EVs with enriched master molecules would benefit many therapeutic programs as a substitute to direct cell therapy. Based on data from this study, we would suggest immortalized MSC-derived EVs enriched with IDO1 as an appropriate candidate in the modulation of immune-related challenges in many scientific or even pre-clinical studies.

## Materials and methods

### Cell lines and primary cultures

Human immortalized adipose tissue-derived mesenchymal stem cells (hTERT-MSCs) were kindly provided by Prof. Steven A. Jonsen (University Medical Center of Göttingen, Germany) and cultured in Dulbecco's modified Eagle’s medium/Ham's F-12 (DMEM/F12) medium (Gibco, Scotland). These cells were previously established from tissue samples of a 30-year-old female and fully characterized by Tatrai et al.^126^. Jurkat cells (Pasteur Institute, Tehran, Iran) were characterized using anti-human candidate antibodies (Supplementary Table S4). HEK293T cells and Jurkat cells were cultured in DMEM-high glucose and Roswell Park Memorial Institute (RPMI) 1640 medium (Gibco, Scotland), respectively. During cell culture procedures, Fetal Bovine Serum (FBS) was applied as the nutritional supplement (10% V/V). Human peripheral blood mononuclear cells (PBMCs) were prepared as primary cultures based on the ethical guidelines of the National Institute for Medical Research Development, which are in accordance with the World Medical Association Declaration of Helsinki. All experimental protocols were approved by the Ethics Committee of NIMAD (ethics code: IRNIMADREC1396040). PBMCs were prepared freshly (in K3EDTA tubes) from healthy volunteers with written informed consent. PBMCs were characterized with single-color antibodies against CD45, CD3, and CD8 (*Table S4*).

### Priming and genetic engineering approaches

#### Cell conditioning and viability test

HTERT-MSCs (10,000 cells/well) were treated with human recombinant interferon-gamma (250 U/ml and 500 U/ml; Type II, > 98% purity, RIFNG50, Invitrogen, USA), and polyinosinic: polycytidylic acid (10 µg/ml and 20 µg/ml; poly(I:C), P1530, Sigma-Aldrich, USA) for 24 h to 72 h. Furthermore, hTERT-MSCs were treated with 10 µg/ml (72 h) of human recombinant GRP78 (BiP) full-length protein (StressMarq, Biosciences, SPR-107). IFN-γ and poly(I:C) primed hTERT-MSCs were subjected to MTT (3-(4, 5-dimethylthiazol-2-yl)-2,5-diphenyltetrazolium bromide; Sigma-Aldrich, Germany, final concentration 500 µg/ml) assay to assess the viability of the cells.

#### Lentiviral gene transfer

Lentiviral vectors including pLenti-CMV-GFP-2A-Puro-Blank as control (LV590), and pLenti-CMV-GFP-2A-Puro containing either of the *IDO1* (LV186089), *PTGS2* (LV276675), and *TGF-β1* (LV333458) insert sequences were purchased from ABM, USA. HEK293T cells were transfected^127^ in the presence of dR8.91 and pMD2.G plasmids. Viral particles were enriched (80-folds) following centrifugation, filtration, and ultracentrifugation (at 70,000*g* for 1 h, fixed-angle rotor type 50.2 Ti rotor, K-factor 69, Optima ultracentrifuge, Model L-90K, Beckman, USA) steps. Different viral constructs (*IDO1*, *PTGS2*, and *TGF-β1*) were introduced to the hTERT-MSCs individually or in combination with each other in the co-transduced group. Non-transduced cells and cells transduced with the blank vector (hTERT-MSCs-GFP) were considered as controls throughout the experiments. HTERT-MSCs successfully transduced with the target constructs were selected based on their acquired resistance to puromycin (1 µg/ml to 2 µg/ml; 48 h, BioBasic, Canada) and were applied for downstream applications on days 5 to 7 post-transduction.

### Immunocytochemistry and Western blot analyses

Immunocytochemistry (ICC) analysis was performed using 25,000 cells/well in 24 well plates (Sarstädt, Germany). Fixation (4% PFA, 20 min), washing (1X PBS), permeabilization (0.25% Triton X-100, 5 min without shaking), blocking (3% BSA, 0.25% Triton X-100, normal donkey serum in 1X PBS), and staining steps were performed 72 h post modifications, except for the negative controls. The cell nuclei were also stained with the fluorescent dye DAPI (Applichem, USA, Cat. No. A1001.0100). To do Western blot experiments against IDO1, PTGS2, and TGF-β1 (Supplementary Table S4), we prepared cell lysis by RIPA buffer (Cell Signaling Technology, USA) and PMSF (Cell Signaling Technology, USA), based on the instructions provided by the manufacturers.

### Differentiation capacity assay

hTERT-MSCs were subjected to osteogenic differentiation in their normal state or following concurrent transduction with all investigated viral particles including *IDO1*, *PTGS2* and *TGF-β1* in the hTERT-MSCs-co-trans group. IFN-γ (250 U/ml) and poly(I:C) (20 µg/ml) were constantly present in the medium in case of differentiation induction procedure for primed cells. In order to induce osteogenic differentiation, β-glycerol phosphate disodium salt hydrate (Sigma-Aldrich, G9422-100, 100x), ascorbic acid (Sigma-Aldrich, 49752, 1000x) and dexamethasone (1000x) (dissolved in ddH_2_O) stock solutions were added to the complete the culture medium with final concentrations of 10 mM (10 µl/ml), 0.5 mM (1 µl/ml), and 0.1 µM (1 µl/ml), respectively. The medium exchange was performed every 48 h for 3 weeks before staining with alizarin red S.

### Molecular studies

Gene expression analyses (*IDO1*, *IDO2*, *TDO2*, *PTGS2*, and *TGF-β1*) were performed on cDNA samples prepared from hTERT-MSCs treated with different concentrations of IFN-γ (250 U/ml, 500 U/ml) and poly(I:C) (10, 20 µg/ml) for 24 to 72 h. The expression levels of a set of immune-encountered genes were investigated for IFN-γ (250 U/ml, 48 h) and poly(I:C)(20 µg/ml, 48 h) treated cells, in addition to the cells primed with BiP (10 µg/ml, 72 h). Moreover, the hTERT-MSCs, which were transduced with different viral particles, including the *IDO1*, *PTGS2*, and *TGF-β*1 individually or in combination, were applied for gene expression analyses on day 7 post-infection. To compare different treatments, we did gene expression analysis for all the genes described in the Supplementary Table S5 in case of the primed or genetically engineered cells, but only genes with significant expression modifications are presented. TRIzol reagent (Ambion, USA) was applied for total RNA extraction. The RNA integrity and quantity were checked and measured by electrophoresis on agarose gel (1%) and using the Nanodrop 2000 spectrophotometer (Thermo Scientific, Wilmington, USA), respectively. One microgram of the DNase I-treated RNA was then applied for cDNA synthesis (all from Thermo Fisher Scientific, USA) in all cases. Relative gene expression analyses were performed using 2 × real-time PCR Master Mix Green-No Rox (Ampliqon, Denmark; CFX-96 Thermocycler, Bio-Rad, USA). Data analysis was performed by CFX Manager Software (version 1.6, Informer Technologies, Inc) and GraphPad Prism version 6.00 (GraphPad Software, La Jolla California USA, www.graphpad.com). Primer sequences (Supplementary Table S5) were selected from previously published articles or otherwise designed by AlleleID 6.0 (Premier Biosoft, Palo Alto, CA, USA, http://www.premierbiosoft.com).

### EVs enrichment and characterization experiments

#### EVs isolation and purification

Culture media of hTERT-MSCs (T175 flasks; seeding density of 1 million cells/flask) were administered to enrich EVs following priming or transduction of the cells. Cells were cultured in the presence of exosome-depleted serum (one-shot fetal bovine serum, exosome depleted, A2720803, Thermo Fisher Scientific, USA) for two rounds of 72 h. It was followed by the pre-clearing steps (centrifugation at 400*g* for 6 min, 1500*g* for 30 min at 4 °C; syringe filtration with 0.45 µm and 0.22 µm filters), which were performed to remove dead cells, cell debris, and intact organelles^128^. The ultracentrifugation method was applied for the enrichment of EVs (70,000*g* at 4 °C for 1 h, fixed-angle rotor type 50.2 Ti rotor, K-factor 69, Optima ultracentrifuge, Model L-90K, Beckman, USA)^129^. Supernatants from the ultracentrifugation were collected in clean tubes, and the remained particles were enriched by the PEG-precipitation method^130^. Besides, EV isolation was also performed by the commercial Exo-spin Kit (EX01, CellGS). The Pierce BCA Protein Assay Kit (Thermo Fisher Scientific, USA) was then applied to measure the total protein content of EV preparations. All relevant data of our experiments, which were in accordance with the ISEV guidelines^131,132^, were submitted to the EV-TRACK knowledgebase (EV-TRACK ID: 190081)^133^.

#### Nanoparticle tracking analysis

Size distribution and the number of particles/ml in each sample (1:100) were measured by a Nanosight NS300 (Malvern Instrument, Malvern, UK) equipped with a 405 nm laser. A 30 s video was automatically recorded for each sample (detection threshold 10 Multi). The processed data were automatically obtained as a PDF file (Nano Tracking Analysis (NTA) version 2.3).

#### Dynamic light scattering and zeta potential analyses

Fresh EV preparations (diluted 1–20 in PBS) were loaded onto the Particle Size Analyzer Cordouan (Vasco3, France). Zeta potential analysis was performed for all the EV samples, diluted in deionized water (1–80) (Zeta Potential, Zeta Compact, CAD, France).

#### Atomic force microscopy (AFM)

To perform AFM, we loaded 1 µl of diluted EV preparations (1:200) onto the freshly cleaved mica and air-dried at room temperature. Imaging was performed in non-contact mode with silicon probe (Tip thickness 10 nm, scan size range 3 µm × 3 µm) covered with aluminum with a scan rate of 3 lines per second. Force spectroscopy was also performed with scan size ranges from 0 to 100 nm in both directions. 4 points with 5 repeats were investigated per sample^134,135^.

#### Western blot

Western blot analysis was performed to verify the expression of widely accepted exosomal markers, including CD63 (Biorbyt, orb11317; 1:500) and TSG101 (Genetex, GTX70255; 1:500). Goat anti-rabbit IgG-HRP (Santa Cruz, sc-2004) and goat anti-mouse IgG-HRP (Santacruz, SC-2005) were applied as secondary antibodies, respectively. Primary mouse monoclonal IgG1 anti-beta Actin antibody [AC-15] (abcam, AB6276) was used as an internal control. EVs (25 µg) were directly diluted 1 to 4 in sample buffer and were applied for western blot experiments following 6-min denaturation at 95 °C. SuperSignal West Femto Kit, Thermo Fisher Scientific, USA) was applied for signal detection.

#### Transmission electron microscopy

EV samples were negatively stained with 2% uranyl acetate and applied for microscopic characterization following Rikkert’s protocol with minor modifications^136^. A similar procedure was applied for the PEG-concentrated ultracentrifuge supernatants, except that samples were diluted (fivefold) in deionized water.

### Functional analysis

#### Lymphocyte inhibition (Orangu) assay

HTERT-MSCs were co-cultured (6-well format) with human PBMCs in RPMI 1640 medium. 72 h later, cell viability was calculated for suspension cells using an Orangu colorimetric assay (Orangu dye, cell counting solution, OR01-500, CellGS), and ODs were read at 450 nm. In addition to MSCs, conditioned medium, isolated EVs, as well as intact and concentrated ultracentrifuge supernatants from different groups, were applied for functional analyses against hPBMCs and/or Jurkat cells in the presence of PHA (3 µg/ml). Functional analyses were performed in 24-well plates for all the cell-free products in the presence of 10% exosome-depleted FBS in the media.

#### Cell proliferation analysis by flow cytometry

CellTrace CFSE Cell Proliferation Kit (Invitrogen, Cat No. C34554) was used to investigate cell proliferation along with different generations following co-culture experiments. PBMCs/Jurkat cells were stained with 10 µM CFSE according to the manufacturer’s instructions and were applied to flow cytometric analysis at different time periods (PBMCs/Jurkat: MSCs ratio 10:1). Propidium iodide (PI, Sigma-Aldrich) staining was also applied to remove dead cells (PI^+^ cells) from the analysis. Functional analyses were performed in the presence of exosome-depleted FBS. In the case of EVs, freshly prepared hPBMCs (5 × 10^5^ cells/well) stained with CFSE (10 µM), were co-cultured with EVs (50 µg/well) enriched from the conditioned media of virally transduced or IFN-γ-primed cells for 5 days (Exosome Isolation Kit, CellGS, UK).

### Statistical analyses

We statistically compared the investigated variables, including cell viability and gene expression levels among different treatment schedules, using a one-way analysis of variance (ANOVA) and multiple comparisons. Each variable is reported as the mean of three independent experiments ± standard error of the mean (SEM). Differences between groups were considered statistically significant at a *p* value of < 0.05. Statistical analyses were performed with GraphPad Prism version 6.00.

## Supplementary Information

Supplementary Information.

## Data Availability

The data used to support the findings of this study are included in the main article or in the Supplementary information files. In case of each analysis, instruments, technical details, sample preparation steps, number of biological or technical repeats, and special software applied for data analysis are also reported. Commercial lentiviral plasmids, reagents and chemicals, cat numbers and providers are mentioned in the manuscript. All relevant data of our experiments were submitted to the EV-TRACK knowledgebase. You may access and check the submission via the following URL: http://evtrack.org/review.php. Please use the EV TRACK ID (EV 190081) and the last name of the first author (Haghighitalab) to access our submission. Data not shown were submitted as Supplementary information (Supplementary figures for reviewers). More detailed information, including pictures, videos, automatic PDF or Excel reports, Figures and curves provided by applied instruments as well as raw data will be available from the corresponding author upon reasonable request.
