# Supplementary material for: Investigating the effects of IDO1, PTGS2, and TGF-β1 overexpression on immunomodulatory properties of hTERT-MSCs and their extracellular vesicles
Source: Sci Rep. 2021 Apr 9;11:7825. doi: 10.1038/s41598-021-87153-7 (PMC8035148; doi:10.1038/s41598-021-87153-7)
Supplement: Supplementary file 1 — Supplementary Information. [file 41598_2021_87153_MOESM1_ESM.pdf]

Supplementary Materials

Original article

Investigating the effects of *IDO1, PTGS2,* and *TGF-β1* overexpression on immunomodulatory properties of hTERT-MSCs and their extracellular vesicles

Azadeh Haghighitalab[1], Maryam M. Matin[1,2], Ahmad Amin[3], Shima Minaee[4], Hamid Reza Bidkhori[5], Thorsten R. Doeppner[6#], Ahmad Reza Bahrami[1,7#]*

[1] Department of Biology, Faculty of Science, Ferdowsi University of Mashhad, Mashhad, Iran

[2] Novel Diagnostics and Therapeutics Research Group, Institute of Biotechnology, Ferdowsi University of Mashhad, Mashhad, Iran

[3] Rajaie Cardiovascular, Medical and Research Center, Iran University of Medical Sciences, Tehran, Iran

[4] Department of Cardiovascular Diseases, Razavi Hospital, Mashhad, Iran

[5] Stem Cells and Regenerative Medicine Research Group, Academic Center for Education, Culture and Research (ACECR)-Khorasan Razavi, Mashhad, Iran

[6] Translational Stroke Research Group, Department of Neurology, University Medical School of Göttingen, Göttingen, Germany

[7] Industrial Biotechnology Research Group, Institute of Biotechnology, Ferdowsi University of Mashhad, Mashhad, Iran

***Corresponding Author:** Correspondence should be addressed to A.R.B. (ar-bahrami@um.ac.ir); Ferdowsi University of Mashhad, Mashhad, Khorasan Razavi, Iran; Telefax: +98 513 8802000.

# Authors contributed equally to the study.

Supplementary Tables

Table S1: Comparative biophysical analysis for EV preparations enriched from the conditioned media of different groups as obtained through zeta potential experiments (Zeta Potential, Zeta Compact, CAD, France).

**Mean ± SEM**	**GFP-EVs**	**IDO1-EVs**	**PTGS2-EVs**	**TGF-β1-EVs**	**co-trans-EVs**	**IFN-EVs**
**Temperature (ºC)**	24.08±0.04	24.86±0.02	24.57±0.04	24.73±0.02	24.46±0.02	24.66±0.05
**Electric Field (V/cm)**	8.42±0.01	8.60±0.0	8.55±0.0	8.61±0.0	8.46±0.0	8.62±0.0
**Conductivity (mS/cm)**	0.67±0.0	0.70±0.0	0.76±0.0	0.66±0.0	0.59±0.0	0.69±0.0
**Dielectric Constant**	78.74±0.02	78.45±0.0	78.56±0.01	78.50±0.0	78.60±0.01	78.53±0.02
**Viscosity (mPa)**	0.90±0.0	0.89±0.01	0.89±0.0	0.89±0.0	0.90±0.0	0.89±0.0
**Mobility (µm/s/V/cm)**	-1.55±0.20	-1.59±0.07	-1.67±0.15	-1.69±0.11	-1.17±0.06	-1.46±0.17
**Zeta Mean (mV)**	-20.26±2.68	-20.50±1.01	-21.61±2.05	-21.84±1.51	-15.23±0.94	-18.90±2.32

Table S2: Data obtained from colorimetric (Orangu dye) lymphocyte inhibition experiments (co-culture of hTERT-MSCs from different groups with human PBMCs or Jurkat cells).

	GFP	IDO1	PTGS2	TGF-β1	Co-trans	Un.	IFN-γ 250	Pol20
**Cells-Jurkat**	100± 0/17	80/06± 0/09	81/47± 0/20	98/10± 0/38	102/51± 0/61	100± 0/14	50/49± 0/20	100/50± 0/17
**Cells-PBMCs**	100± 5/48	69/43± 2/56	70/29± 3/02	62/59± 4/28	35/75± 1/41	100± 0/40	83/26± 2/68	100± 0/38
**C.M. Jurkat**	100± 0/26	101/49± 0/66	100/78± 0/55	100/49± 0/03	101/18± 0/29	100± 5/34	100± 0/03	100± 0/72
**C.M. PBMCs**	100± 0/23	84/43± 0/23	94/93± 0/32	91± 0/14	91/39± 0/09	100± 0/06	100± 0/46	89/48± 1/33

Data represent mean of cell viabilities (%) for PBMCs or Jurkat cells following 72 h co-culture experiments (data are reported as Mean ± SEM) in comparison to controls. Abbreviations: C.M., conditioned medium; Un., untreated hTERT-MSCs.

Table S3: Data from lymphocyte inhibition assay following co-culture experiments among cell free products prepared from hTERT-MSCs cultures under different circumstances and human PBMCs or Jurkat cells (colorimetric Orangu dye).

Time	Group	PB/JU	GFP	IDO1	PTGS2	TGF-β1	Co-trans	Un.	IFN-γ 250	Pol20
**72 h**	**Exosomes**	100± 1.15	54.58± 0.03	55.03± 0.00	100± 0.69	88.70± 11.09	70.38± 0.46	62.13± 0.81	67.25± 0.09	65.41± 0.38
**96 h**	**Exosomes**	100± 3.06	35.47± 1.18	30.67± 0.69	35.72±5 .40	30.80± 6.64	37.00± 2.28	56.67± 0.00	64.72± 0.95	59.08± 0.12
**72 h**	**Concentrated UC. Sup**	100± 0.46	71.60± 0.06	63.74± 0.03	67.63±0 .12	69.54± 0.49	62.74± 0.12	91.83± 0.43	87.78± 0.17	88.09± 1.44
**96 h**	**Concentrated UC. Sup**	100± 3.06	63.92± 0.26	49.93± 2.17	58.44±0 .12	59.53± 0.61	49.61± 7.68	91.28± 1.67	89.93± 1.73	85.91± 1.88
**72 h**	**UC. Sup**	100± 3.84	100± 0.29	79.27± 0.06	100± 0.17	100± 3.29	89.34± 2.74	94.27± 5.51	95.98± 6.12	83.92± 5.95
**96 h**	**UC. Sup**	100± 3.44	89.43± 0.87	91.45± 5.11	100.27± 1.67	93.16± 7.01	97.13± 4.42	105.93± 1.53	100.10± 2.11	98.32± 2.02

Data represent mean of cell viabilities (%) for PBMCs or Jurkat cells following 72 h and 96 h co-culture experiments in comparison to controls (data are reported as Mean ± SEM). Abbreviations: JU, Jurkat cells; PB, PBMCs; Sup, supernatant; UC., ultracentrifuge: Un., untreated hTERT-MSCs.

Table S4: Primary and secondary antibodies used in the current study.

No.	Antibody	Primary/ Secondary	Application	Provider/Country	Isotype/Clone	Cat No.
1	APC-conjugated anti-human CD45	Single-color	FC	CYTOGNOS/ Spain	IgG1 (HI30)	CYT-45AP5
2	PE-Cy 5-conjugated anti-human CD3	Single-color	FC	CYTOGNOS/ Spain	IgG1 (UCHT-1)	CYT-3C4
3	FITC-conjugated anti-human CD4	Single-color	FC	CYTOGNOS/ Spain	IgG2a (Edu-2)	CYT-4F6
4	PE-conjugated anti-human CD8	Single-color	FC	CYTOGNOS/ Spain	IgG2a (UCHT-4)	CYT-8PE9
5	APC-conjugated-anti-CD44	Single-color	FC	BD-Biosciences/EU	Mouse IgG_{2b}, κ	559942
6	PE-conjugated-anti-CD73	Single-color	FC	BD-Biosciences/EU	Mouse IgG1, κ	561014
7	PE-conjugated-anti-CD39	Single-color	FC	BD-Biosciences/EU	Mouse IgG2b, κ	555464
8	FITC-conjugated-anti-HLA-DR	Single-color	FC	BD-Biosciences/EU	Mouse IgG2a, κ	562008
9	PE-conjugated-anti-CD11b	Single-color	FC	BD-Biosciences/EU	Mouse BALB/c IgG2a, κ	333142
10	PE.Cy5-conjugated-anti-CD19	Single-color	FC	CYTOGNOS/Spain	IgG1 (HIB19)	CYT-19C3
11	Rabbit anti-hIDO1	Primary	ICC/WB	Cell Signaling Technology/USA	IgG	86630
12	Goat anti-hPTGS2	Primary	ICC/WB	R&D Systems/USA	IgG	AF4198
13	Mouse anti- hTGF-β1	Primary	ICC/WB	AB Clonal/USA	IgG	A0291
14	Donkey anti-rabbit	Secondary	ICC	Jackson Immuno/UK	IgG (H+L)	711-547-003
15	Donkey anti-goat	Secondary	ICC	Jackson Immuno/UK	IgG	705-165-003
16	Donkey anti-mouse	Secondary	ICC	Jackson Immuno/UK	IgG	115-165-164
17	Goat anti-rabbit IgG-HRP	Secondary	WB	Santa Cruz/Germany	IgG	sc-2004
18	Donkey anti-goat IgG-HRP	Secondary	WB	Santa Cruz/ Germany	IgG	sc-2020
19	Goat anti-mouse IgG-HRP	Secondary	WB	Santa Cruz/ Germany	IgG	sc-2005

Table S5: Real-time specific primer sequences.

No.	Gene name	Tm	Sequence (5' to 3')	Length (bp)	Ref.
1	***DDX58***<br>Refseq Access. NM 014314.4	60	**F**: TGTGCTCCTACAGGTTGTGGA<br>**R**: CACTGGGATCTGATTCGCAAAA	120	(Zhao, *et al.*, 2013)
2	***HLA-DR***<br>Refseq Access. NM 001243965.1	60	**F**: AGCGGCGAGTCCATCCTAAG<br>**R**: ACACCACCCCAGTCTTCTCTTC	156	-
3	***IDO1***<br>Refseq Access. NM 002164.6	60	**F**: TCATCTCACAGACCACAAGTCA<br>**R**: GCAAGACCTTACGGACATCTCC	107	(Adams, *et al.*, 2014)
4	***IDO2***<br>Refseq Access. NM 194294.2	60	**F**: TGCTTCATGCCTTTGATGAG<br>**R**: GAAGGCCTTATGGGAAGGAG	104	(Opitz, *et al.*, 2011)
5	***IFIH1***<br>Refseq Access. NM 022168.4	60	**F**: TCGAATGGGTATTCCACAGACG<br>**R**: GTGGCGACTGTCCTCTGAA	152	(Zhu, *et al.*, 2017)
6	***IFN-β1***<br>Refseq Access. NM 002176.4	60	**F**: GCTTGGATTCCTACAAAGAAGCA<br>**R**: ATAGATGGTCAATGCGGCGTC	166	(Lio, *et al.*, 2016)
7	***IFN-γ***<br>Refseq Access. NM 000619.3	60	**F**: CTTTAAAGATGACCAGAGCATCCA<br>**R**: GGCGACAGTTCAGCCATCAC	189	-
8	***IL-10***<br>Refseq Access. NM 000572.3	60	**F**: GAGATGCCTTCAGCAGAGTGAAGA<br>**R**: AGGCTTGGCAACCCAGGTAAC	114	-
9	***IL-6***<br>Refseq Access. NM 000600.5	60	**F**: ACTCACCTCTTCAGAACGAATTG<br>**R**: GCAAGTCTCCTCATTGAATCCAG	196	-
10	***MYD88***<br>Refseq Access. NM 001172567.2	60	**F**: ACTGCTCGAGCTGCTTACCAA<br>**R**:CTCCTGCTGCTGCTTCAAGAT	106	(Feng, *et al.*, 2016)
11	***OAS2***<br>Refseq Access. NM 016817.3	60	**F:** TTCTGCCTGCACCACTCTTC<br>**R:** TCTTCAGAGCTGTGCCTTTG	210	-
12	***PD-L1***<br>Refseq Access. NM 014143.4	60	**F**: AAGTCAATGCCCCATACAAC<br>**R**: TGCTTGTCCAGATGACTTCG	123	-
13	***PTGS2***<br>Refseq Access. NM 000963.4	60	**F**: CCCTTGGGTGTCAAAGGTA<br>**R**: GCCCTCGCTTATGATCTGTC	169	(Hou, *et al.*, 2011)
14	***RPLP0***<br>Refseq Access. NM 001002.4	60	**F**: TGGTCATCCAGCAGGTGTTCGA<br>**R**: ACAGACACTGGCAACATTGCGG	119	-
15	***STING***<br>Refseq Access. NM 198282.4	60	**F**: CACTTGGATGCTTGCCCTC<br>**R**: GCCACGTTGAAATTCCCTTTTT	117	(Ribon, 2015)
16	***TDO2***<br>Refseq Access. NM 005651.4	60	**F**: ATTCATAAGGATTCAGGCTAAAG<br>**R**: TTTCTCATCAAATAAGGACAGTA	103	(Adams, *et al.*, 2014)
17	***TGF-β1***<br>Refseq Access. NM 000660.7	60	**F**: AGCGACTCGCCAGAGTGGTTA<br>**R**: GCAGTGTGTTATCCCTGCTGTCA	130	(Sumitomo, *et al.*, 2013)
18	***TLR-3***<br>Refseq Access. NM 003265.3	60	**F**: CAAACACAAGCATTCGGAATCTG<br>**R**: AAGGAATCGTTACCAACCACATT	145	(Kreutz, *et al.*, 2015)
19	***TLR-4***<br>Refseq Access. NM 138554.5	60	**F**: AGTTGATCTACCAAGCCTTGAGT<br>**R**:GCTGGTTGTCCCAAAATCACTTT	94	(Chen, *et al.*, 2016b)

DDX58 (RIG-I: retinoic acid-inducible gene-I): DExD/H-box helicase 58; HLA-DR: Human Leukocyte Antigen, class II, DR beta; IDO1: Indole amine 2,3 dioxygenase 1; IDO2: Indole amine 2,3 dioxygenase 2; IFIH1: Interferon-induced helicase C domain-containing protein 1; IFN-β1: Interferon beta 1; IFN-γ: Interferon gamma; IL-10: Interleukin 10; IL-6: Interleukin 6; MYD88: innate immune signal transduction adaptor; OAS2: 2'-5'-Oligoadenylate synthetase 2; PDL-1 (CD274, B7 Homolog 1): Programmed cell death ligand-1; PTGS2: Prostaglandin-endoperoxide synthase 2; RPLP0: Ribosomal protein lateral stalk subunit P0; STING (TMEM173 transmembrane protein 173): Stimulator of interferon genes; TDO2: Tryptophan 2,3-dioxygenase; TGF-β1: Transforming growth factor beta 1; TLR-3: Toll like receptor 3; TLR-4: Toll like receptor 4.

Supplementary Figures

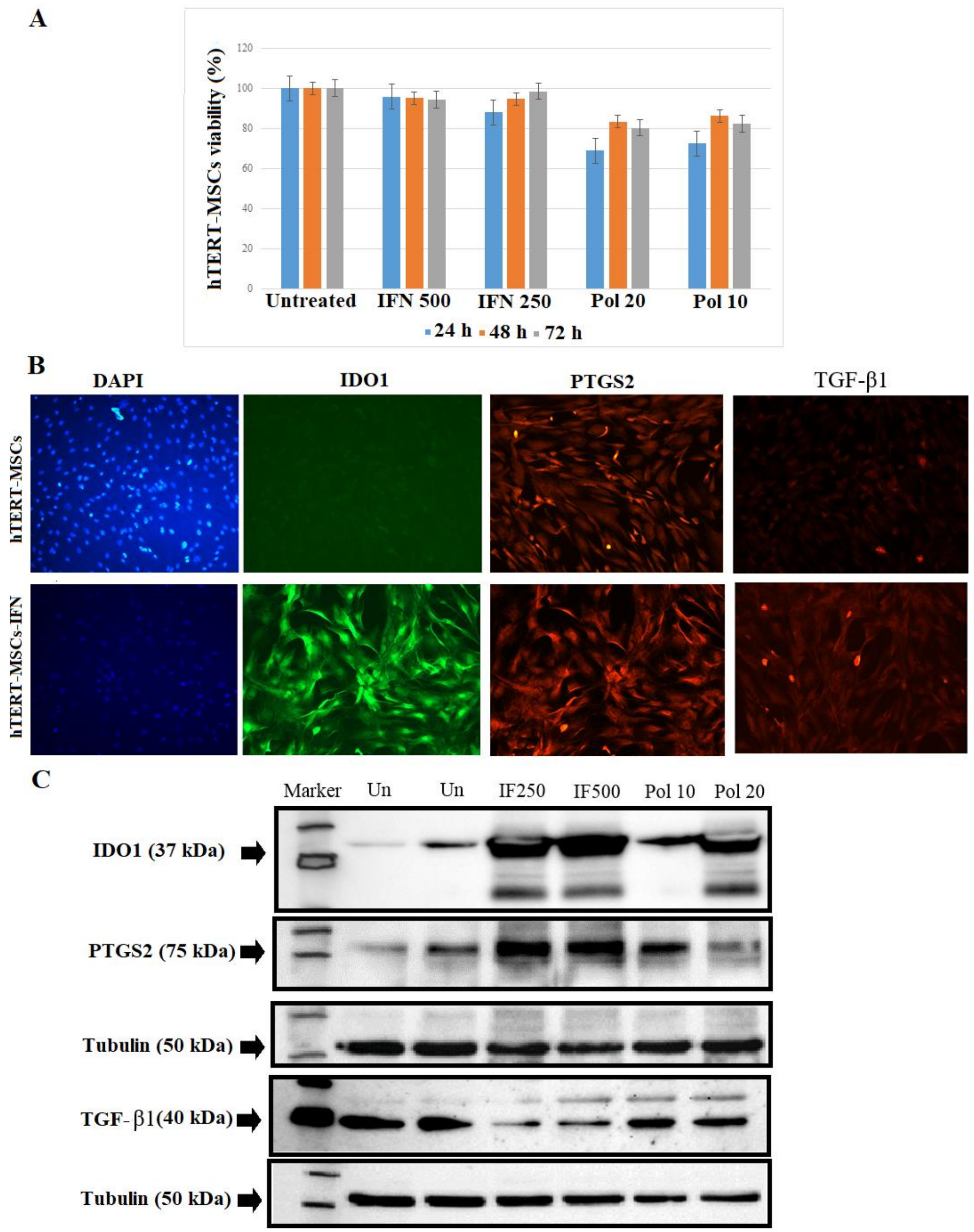

Figure S1. A) Cell viability of hTERT-MSCs following treatment with different concentrations of IFN-γ (250 U/ml, 500 U/ml) and poly(I:C) (10 µg/ml, 20 µg/ml) for 24 h, 48 h and 72 h. Mean of three independent experiments are reported ± SEM in each case (Microplate Reader, Tecan Life Sciences, Switzerland). B) Expression of IDO1, TGF-β1 and PTGS2 was verified by immunocytochemistry following treatment of hTERT-MSCs with IFN-γ (250 U/ml) for 72 h in comparison to untreated cells (Axioplan 2 Fluorescence Microscope, Carl Zeiss, Germany). C)

Protein expression analysis of hTERT-MSCs following treatment with different concentrations of IFN-γ and poly(I:C) for 72 h. α-Tubulin was applied as internal control. ChemiDoc XRS+ (Bio-Rad) imaging system was applied to detect ECL reagent signal. Full-length blots are included in the supplementary Materials (Supplementary figures for reviewers).

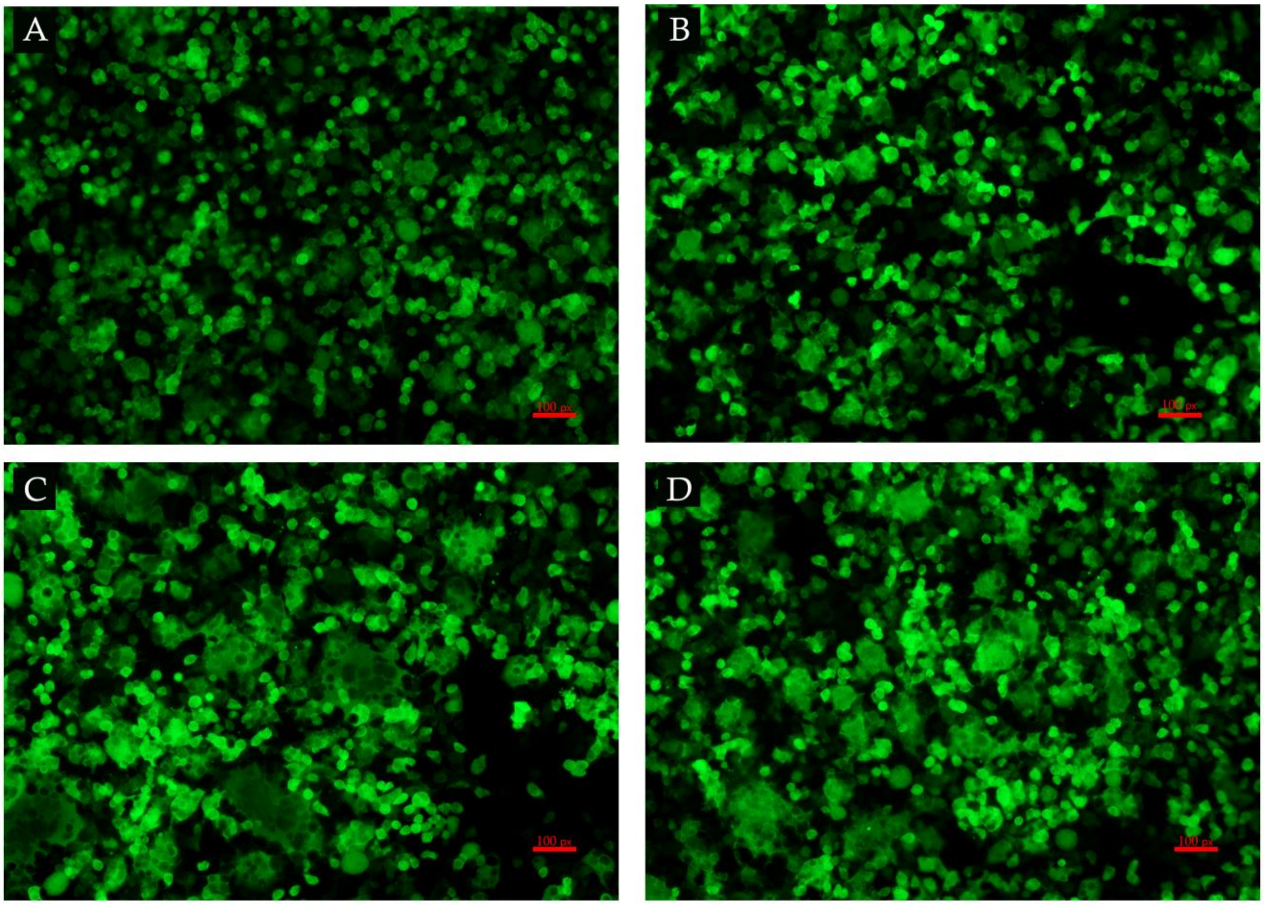

Figure S2. HEK293T cells transfected with target plasmids, carrying *GFP*, *IDO1*, *PTGS2* and *TGF-β1* genes 2 days post transfection (**a** to **d**, respectively, Nikon, Japan, scale bars represent 100 pixel).

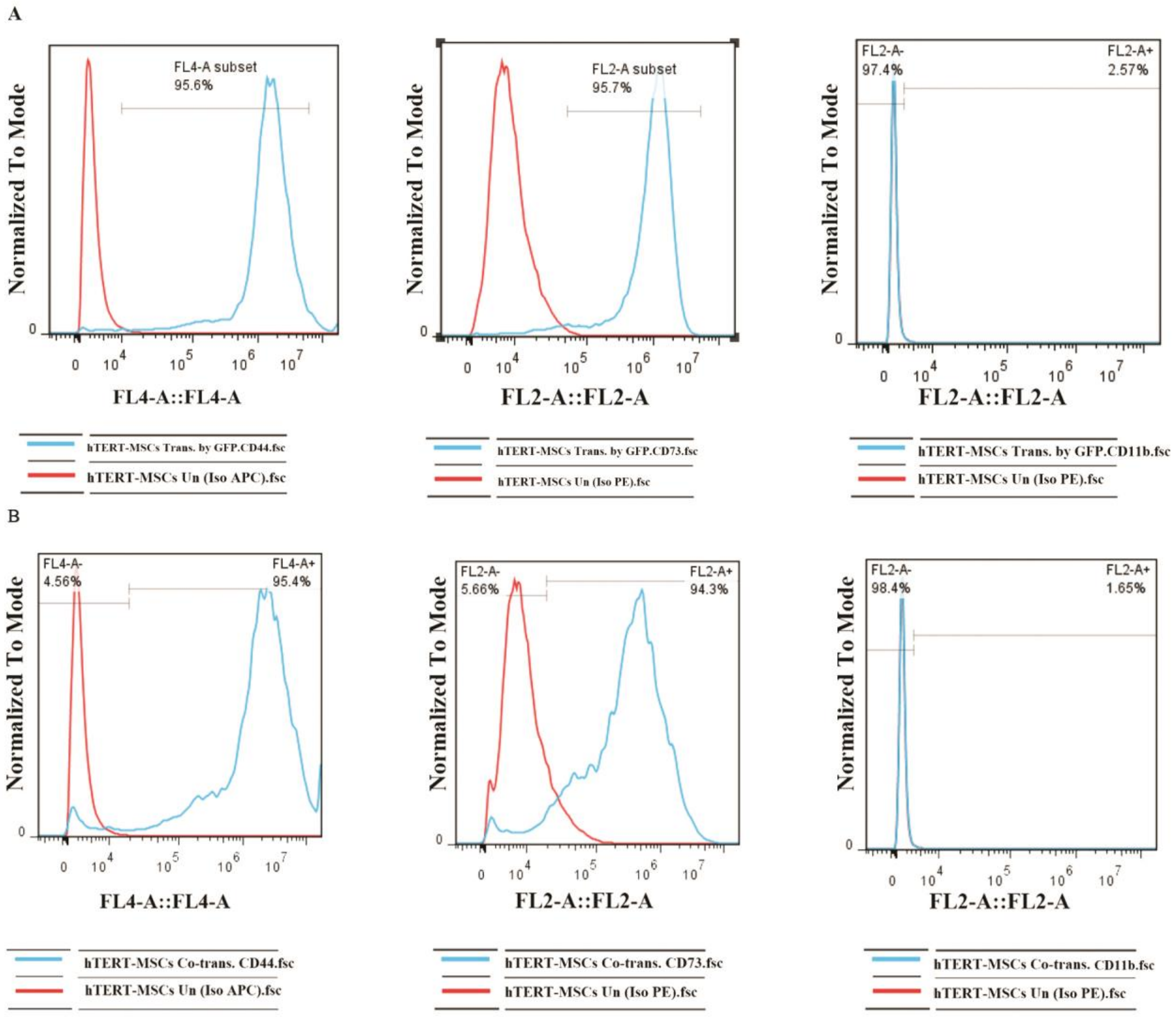

Figure S3. Flow-cytometric immune phenotyping of the hTERT-MSCs following viral transduction with control GFP or co-transduction with all three viral particles. Data were obtained by BD Accuri C6 and analyzed using FlowJo Software (version 7.6).

A B

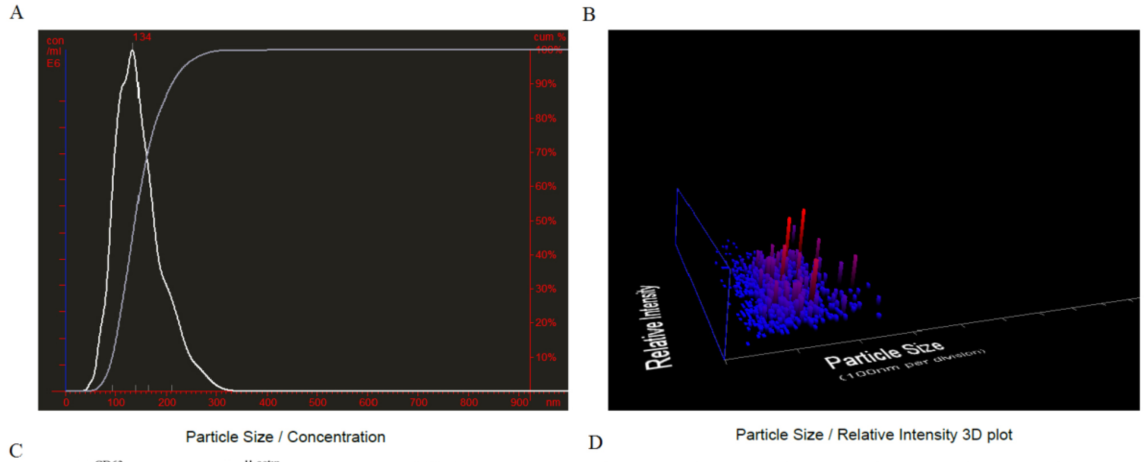

C

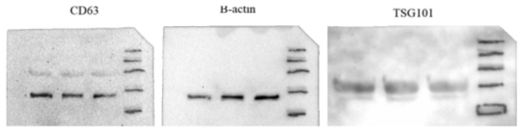

D

Protein concentration of EV samples (BCA assay, µg/ml)					
Un-MSC	IFN-MSC	Pol-MSC	Un-MSC	IFN-MSC	Pol-MSC
1.892	1.946	1.881	1.446	1.352	1.505

Particle/protein ratio					
Un-MSC	IFN-MSC	Pol-MSC	Un-MSC	IFN-MSC	Pol-MSC
8.54E+10	4.99E+10	8.51E+10	7.86E+10	4.65E+10	3.48E+10

E

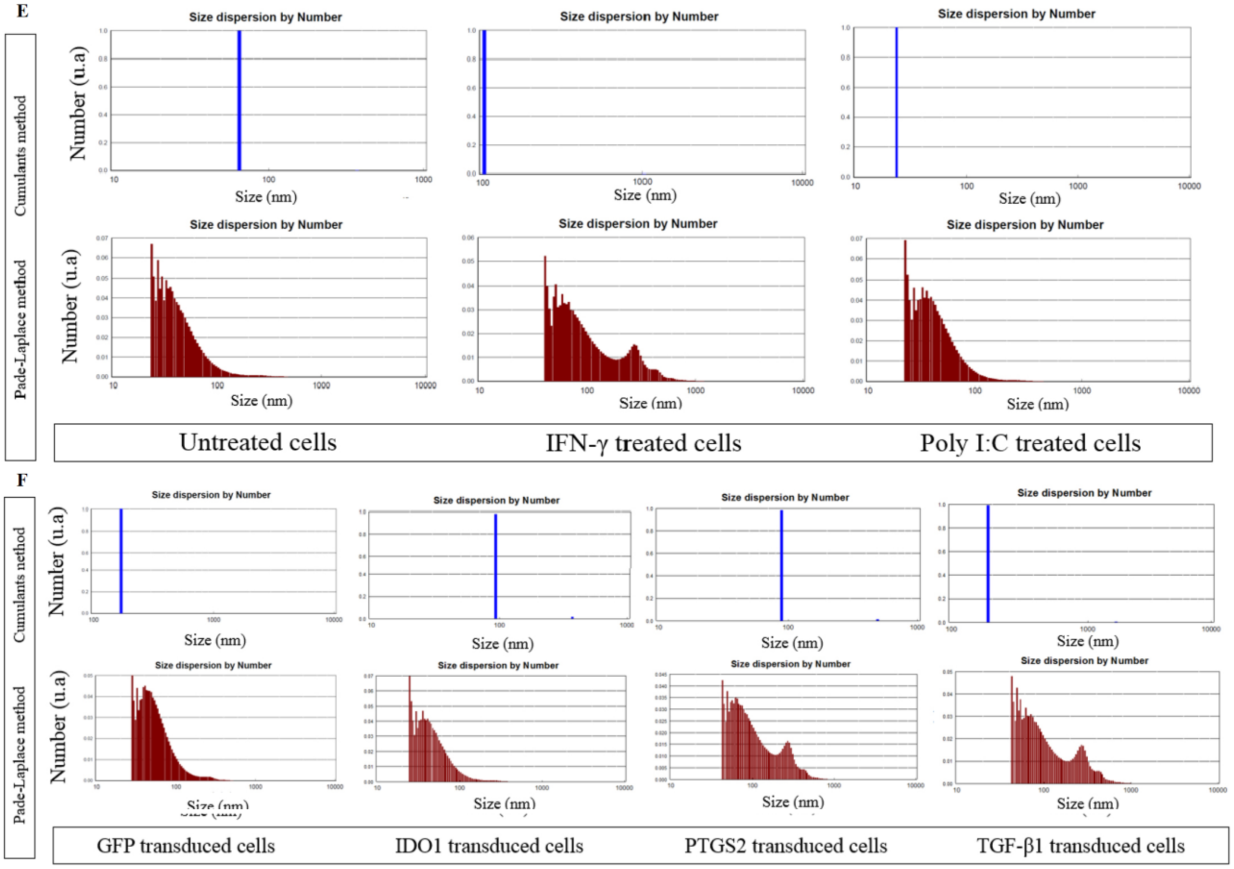

Figure S4. A, B) Nano particle tracking analysis performed for extracellular vesicles (EVs), isolated from conditioned medium of hTERT-MSCs (after 72 h in culture), following centrifugation, filtration, and ultracentrifugation steps. Particle size/concentration and particle size/relative intensity 3D curves are demonstrated for EVs isolated from untreated hTERT-MSCs. Two biological repeats were considered for each treatment and three reports recorded for

each sample during the NPA (Nanosight NS300, Malvern, UK). The processed data were automatically obtained as a PDF file (Nano Tracking Analysis (NTA) version 2.3). No customized value considered for analysis. C) Characterization of the EVs using exosomal markers CD63 and TSG101 using Western blot analysis. EVs were isolated from the untreated and IFN-γ-treated and poly(I:C)-treated cells by ultracentrifugation from 72 h conditioned media of the cells. Full-length blots are included in the supplementary Materials (Supplementary figures for reviewers). Blots were cut prior to the hybridization with primary antibodies. D) Mean of protein concentration (µg/ml) was calculated based on standard BCA assay for different treatments. In addition, particle to protein ratios are reported for different samples. As it is obvious best particle to protein ratio was recorded for untreated cells. E, F) Size dispersion of detected particles during particle size analysis. The number of detected particles versus size (nm) as recorded by DLS device. E) EVs derived from the hTERT-MSCs conditioned with the human recombinant IFN-γ (250 U/ml) or poly(I:C) for 72 h. F) hTERT-MSCs following transduction with different viral particles (GFP, IDO1, PTGS2, TGF-β1 and co-transduced). Data obtained automatically by DLS device based on Cumulants and Pade-Laplace methods.

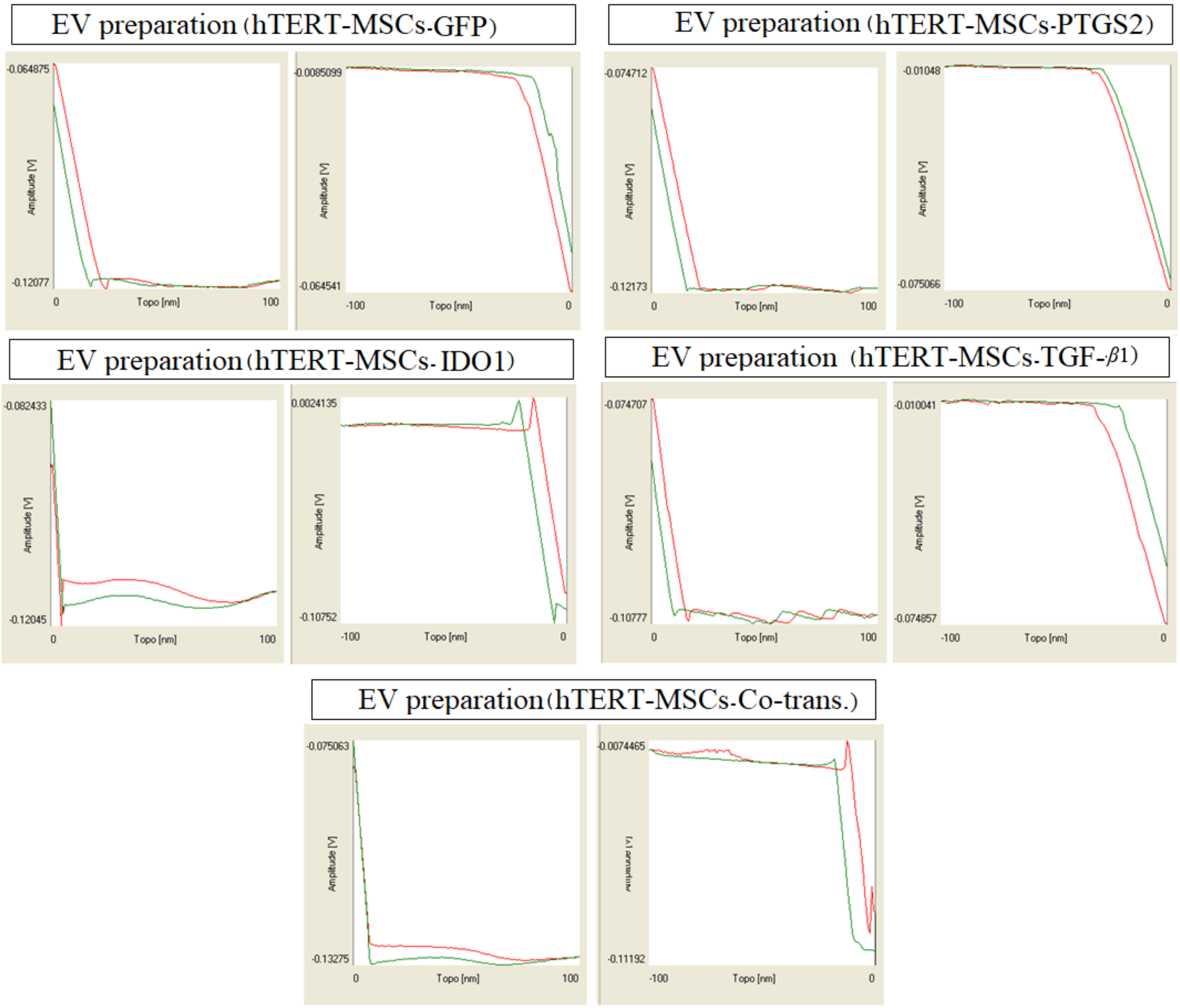

Figure S5. Force spectroscopic analysis via atomic force microscopy. Comparative force spectroscopy experiments performed for extracellular vesicles obtained by ultracentrifugation method from the conditioned media of hTERT-MSCs (resonance frequency 220 mV, 336.982 kHz; Spring constant (NSC15) 40). In each curve the red line indicates force-response curve (approach curve), while the green curve indicates retraction curve. Image processing and analysis were performed by Imager version 1.00 AraResearch Co.

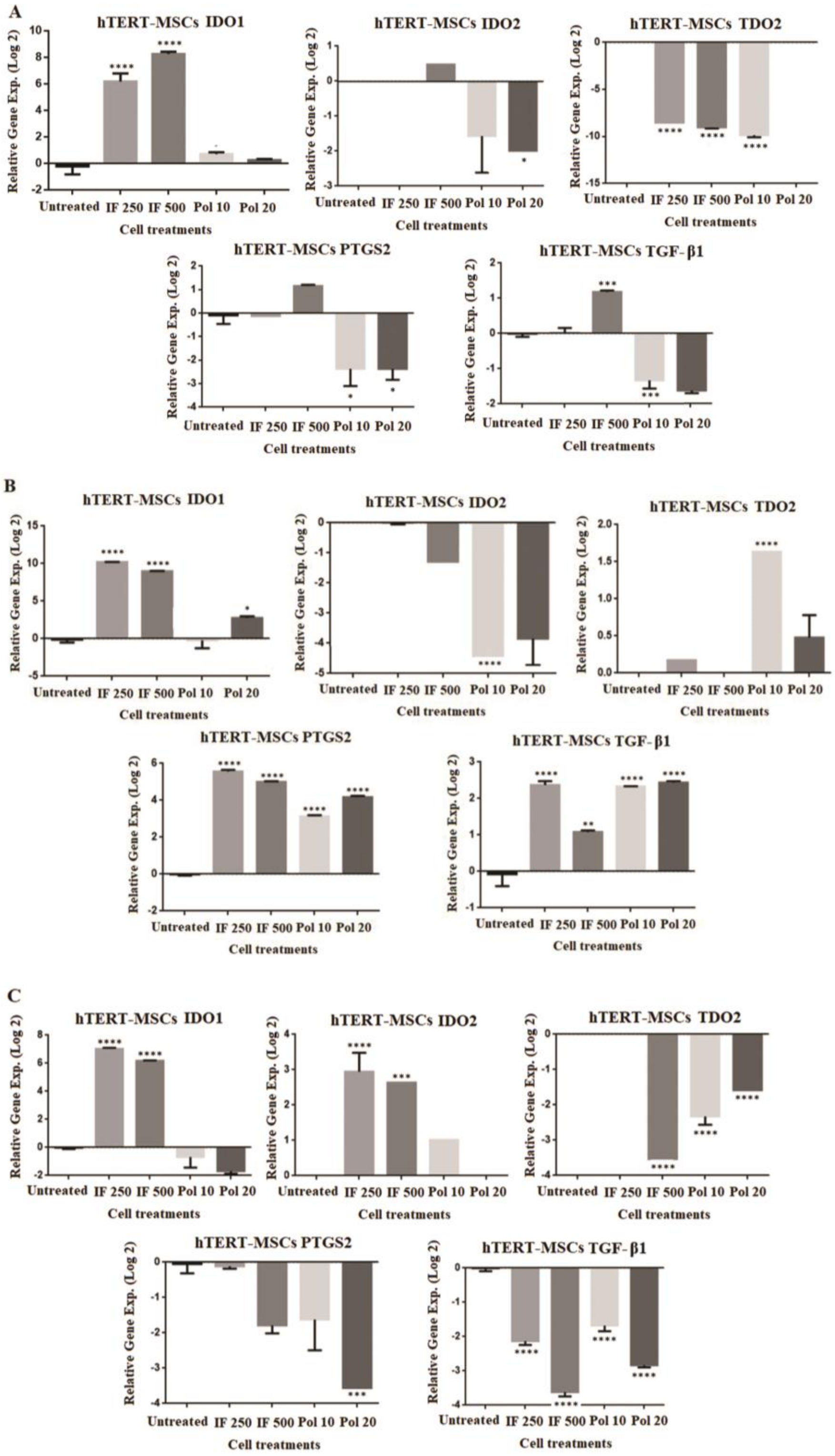

Figure S6. Gene expression analysis for the hTERT-MSCs following treatment with different concentrations of IFN-γ and poly(I:C) for 24 h (A), 48 h (B) and 72 h (C). Data were obtained by qRT-PCR analysis and reported as mean of three independent experiments ± SEM. Gene expression levels for various treatments were compared to untreated cells. RPLP0 was

considered as internal control in all experiments. Data analysis was performed by CFX Manager Software (version 1.6, Informer Technologies, Inc), and GraphPad Prism version 6.00 (GraphPad Software, La Jolla California USA, www.graphpad.com). *$p < 0.05$, **$p < 0.01$, ***$p < 0.001$ and ****$p < 0.0001$.

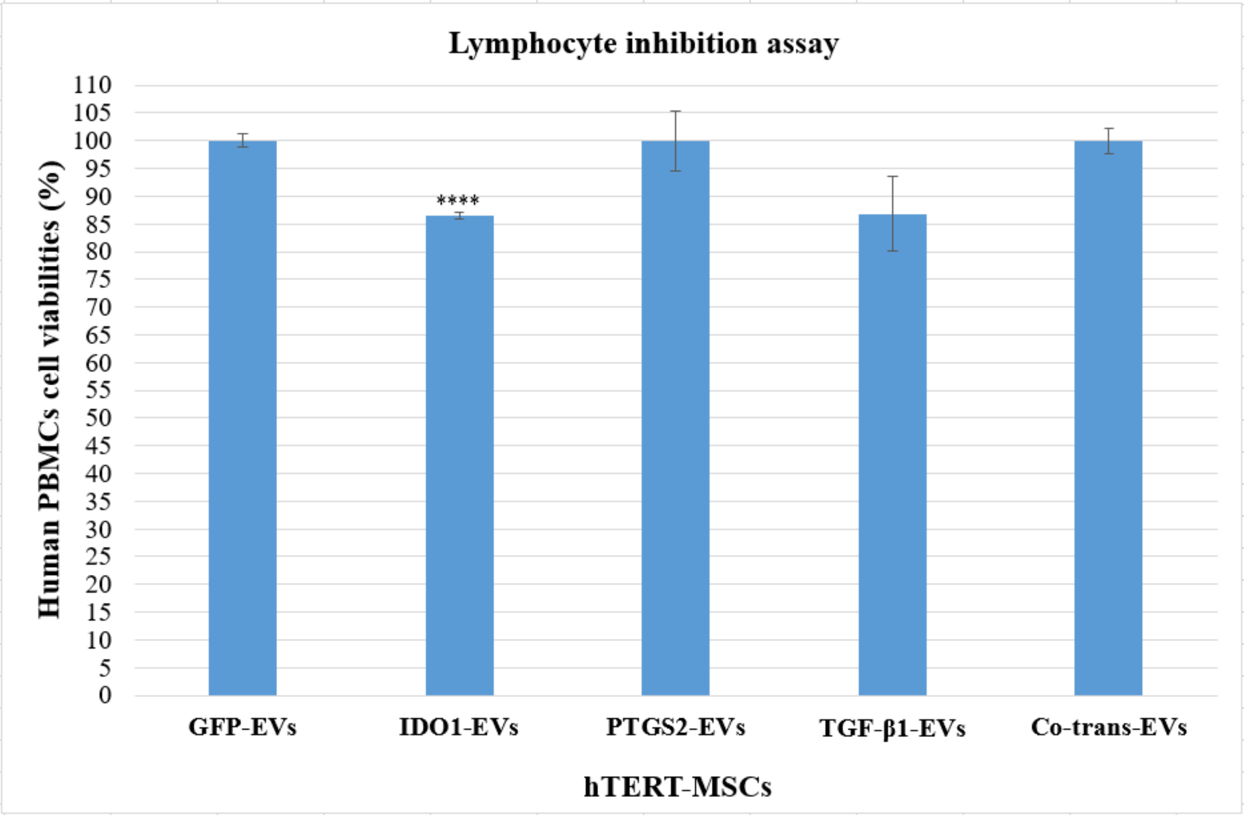

Figure S7. Functional analysis of EVs from genetically modified hTERT-MSCs. Lymphocyte inhibition assay experiments were performed for EVs, in the permanent presence of 3 µg/ml PHA as the PBMCs proliferation stimulant. 96 h co-cultures were applied for functional analysis based on the detection of viable cells via sensitive Orangu dye. 100 µg of EVs from different groups were added to the culture media of hPBMCs (10^6 cells/well). Data represent mean of three independent experiments $\pm$ SEM. Relative ODs (450 nm) in each group were read by an ELISA reader (Awareness Technology, USA) following 4 h incubation. Mean of cell viabilities for GFP-transduced cells were considered equal to 100%. Data analysis was performed by GraphPad Prism version 6.00 (GraphPad Software, La Jolla California USA, www.graphpad.com). ****$p < 0.0001$.

Supplementary Figures for Reviewers

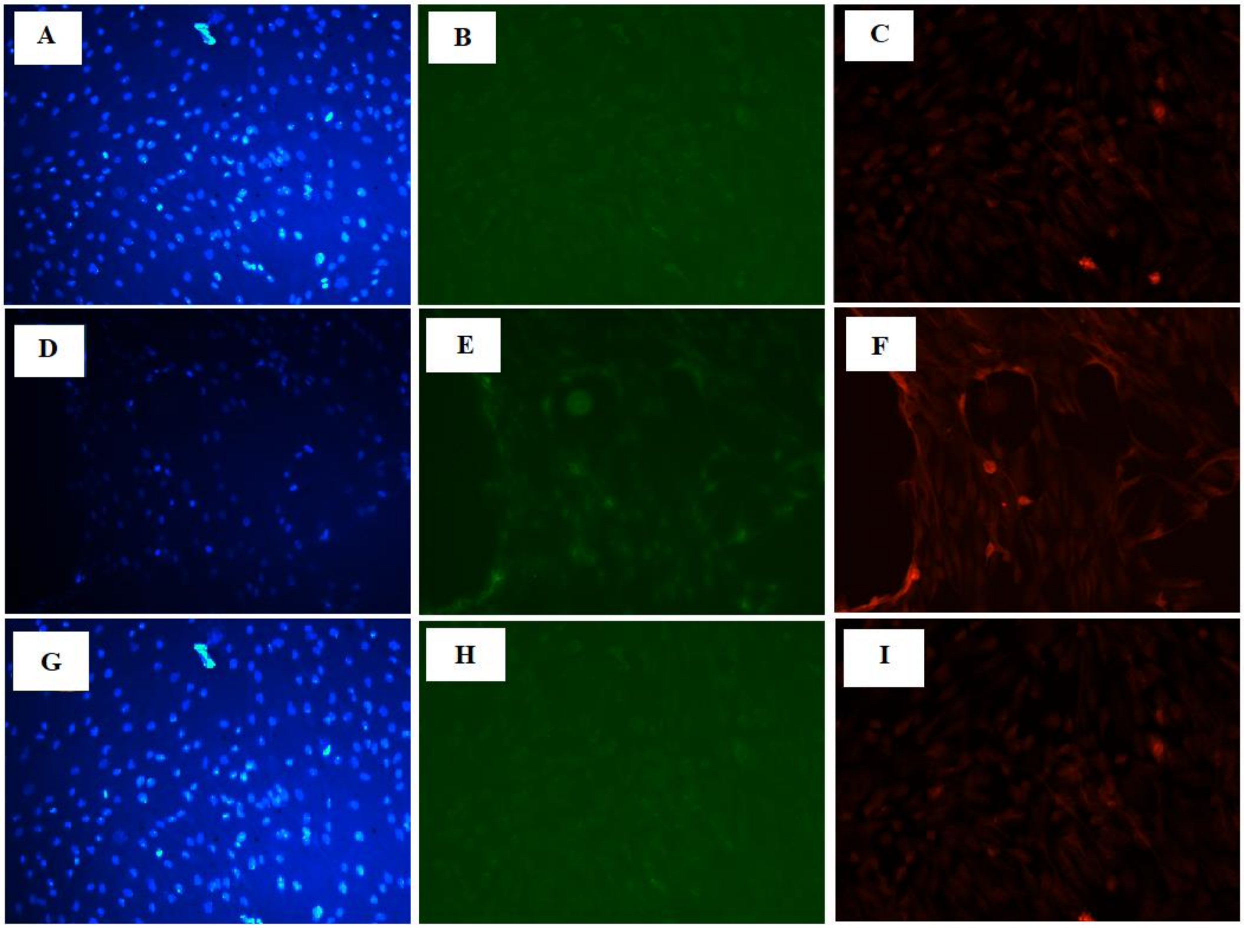

Figure SR1. Immunocytochemistry analysis of IDO1 (Second column) and TGF-β1 (Third column) expression in untreated hTERT-MSCs (first row, A-C) or the cells treated with poly(I:C) 10 µg/ml (second row, D-F) and poly(I:C) 20 µg/ml (third row, G-I). As demonstrated in A, D and G, DAPI staining was applied to detect the nuclear region of the cells during ICC experiments by fluorescent microscopy (Axioplan 2 Fluorescence Microscope, Carl Zeiss, Germany). It is obvious from the results that IDO1 expression in the cells treated with 10 µg/ml of poly(I:C) prominently limited to the nuclear compartment of the cells instead of cytoplasmic regions.

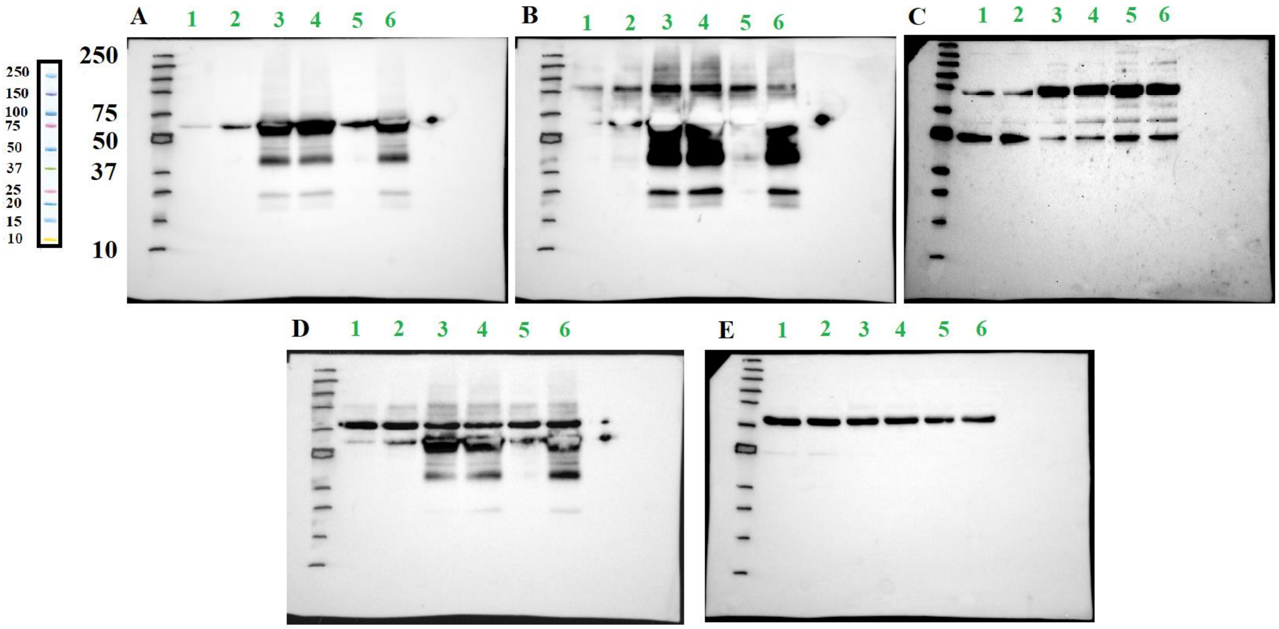

Figure SR2. Full-length blots obtained for different proteins including IDO1 (A), PTGS2 (B), and TGF-β1(C) during western blot analysis. In each part, lines 1-2 indicated untreated hTERT-MSCs, lines 3 and 4 indicated IFN-γ treated cells (250 and 500 U/ml, respectively). Lines 5-6 indicated poly(I:C) treated hTERT-MSCs (10 and 20 µg/ml, respectively). D (for IDO1 and PTGS2) and F (for TGF-β1) represent full-length blots for α- Tubulin which was applied as internal control. The same blot applied for IDO1 detection, was applied to detect COX-2 and Tubulin without stripping, as they have different sizes. ChemiDoc XRS+ (Bio-Rad) imaging system was applied to detect ECL reagent signal.

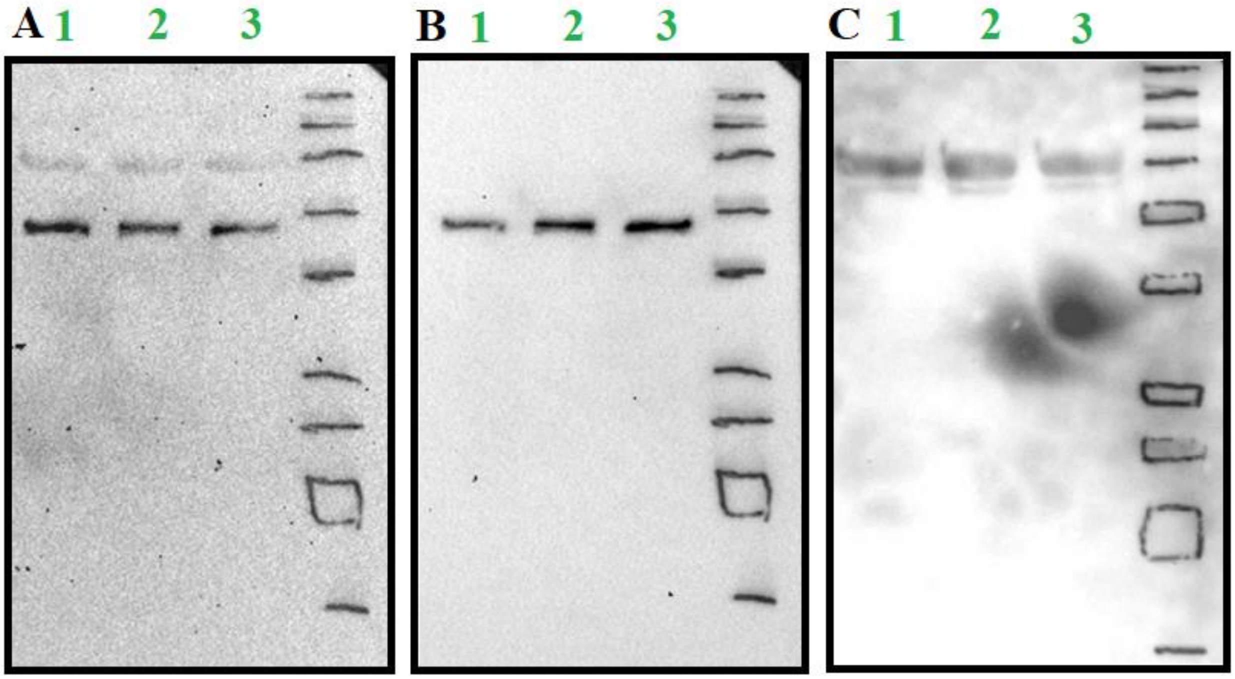

Figure SR3. Full-length blots obtained for common exosomal proteins CD63 (A) and TSG101 (C) in hTERT-MSCs-EVs prepared from poly(I:C)-, IFN-γ-treated, and untreated cells, respectively (lines 1-3 in each blot). (B) represents full-length blot obtained for β- actin which was applied as internal control. Blots were cut prior to the hybridization with different primary antibodies. ChemiDoc XRS+ (Bio-Rad) imaging system was applied to detect ECL reagent signal.
